# Cross-reactivity influences changes in human influenza A virus and Epstein Barr virus specific CD8 memory T cell receptor alpha and beta repertoires between young and old

**DOI:** 10.3389/fimmu.2022.1011935

**Published:** 2023-02-24

**Authors:** Fransenio Clark, Anna Gil, Ishwor Thapa, Nuray Aslan, Dario Ghersi, Liisa K. Selin

**Affiliations:** ^1^ Department of Pathology, University of Massachusetts Medical School, Worcester, MA, United States; ^2^ School of Interdisciplinary Informatics, University of Nebraska at Omaha, Omaha, NE, United States

**Keywords:** influenza A virus (IAV)-M158-66 epitope, Epstein Barr virus (EBV)-BMLF1280-288 epitope, Epstein Barr virus (EBV)-BRLF1109-117 epitope, T cell receptor (TCR) repertoire, crossreactivity, aging

## Abstract

Older people have difficulty controlling infection with common viruses such as influenza A virus (IAV), RNA virus which causes recurrent infections due to a high rate of genetic mutation, and Epstein Barr virus (EBV), DNA virus which persists in B cells for life in the 95% of people that become acutely infected. We questioned whether changes in epitope-specific memory CD8 T cell receptor (TCR) repertoires to these two common viruses could occur with increasing age and contribute to waning immunity. We compared CD8 memory TCR alpha and beta repertoires in two HLA-A2+ EBV- and IAV-immune donors, young (Y) and older (O) donors to three immunodominant epitopes known to be cross-reactive, IAV-M1_58-66_ (IAV-M1), EBV-BMLF1_280-288_ (EBV-BM), and EBV-BRLF1_109-117_ (EBV-BR). We, therefore, also designed these studies to examine if TCR cross-reactivity could contribute to changes in repertoire with increasing age. TCR high throughput sequencing showed a significant difference in the pattern of TRBV usage between Y and O. However, there were many more differences in AV and AJ usage, between the age groups suggesting that changes in TCRα usage may play a greater role in evolution of the TCR repertoire emphasizing the importance of studying TRAV repertoires. With increasing age there was a preferential retention of TCR for all three epitopes with features in their complementarity-determining region (CDR3) that increased their ease of generation, and their cross-reactive potential. Young and older donors differed in the patterns of AV/AJ and BV/BJ pairings and usage of dominant CDR3 motifs specific to all three epitopes. Both young and older donors had cross-reactive responses between these 3 epitopes, which were unique and differed from the cognate responses having features that suggested they could interact with either ligand. There was an increased tendency for the classic IAV-M1 specific clone BV19-IRSS-JB2.7/AV27-CAGGGSQGNLIF-AJ42 to appear among the cross-reactive clones, suggesting that the dominance of this clone may relate to its cross-reactivity with EBV. These results suggest that although young and older donors retain classic TCR features for each epitope their repertoires are gradually changing with age, maintaining TCRs that are cross-reactive between these two common human viruses, one with recurrent infections and the other a persistent virus which frequently reactivates.

## Introduction

CD8 T cell recognition of virus-infected cells requires a specific interaction between short peptides presented by HLA Class I molecules on infected cell surfaces and TCRαβ heterodimers on CD8 T cells. These virus epitope-specific memory CD8 T cells develop complex TCR repertoires that are specific for that epitope. State-of-the-art high throughput and single cell sequencing provide a more unbiased understanding of antigen-specific TCR repertoires. CD8 T cell TCR repertoires to common viruses, IAV, cytomegalovirus (CMV) are highly diverse and individualized i.e. “private” (1). However, despite this diversity there are clonotypes with “public” features, *i.e.* preferential usage of particular variable (V) region or conserved or identical amino acid motifs within the complementarity-determining regions (CDR3α/β) for each epitope that appear to be favored for expansion, likely due to selection for optimal structural interactions (2, 3).

We have been studying TCR repertoires to both IAV and EBV immunodominant epitopes in HLA-A2+ donors, focusing on IAV-M1, EBV-BM and EBV-BR, in order to identify their public characteristics to better understand antigen-specific TCR selection. Our recent results in IAV-immune healthy donors would suggest that the number of contacts between TCR and peptide major histocompatibility complex (pMHC) is a controlling factor in determining TCR selection (3) and that antigen-specific TCR repertoires have evolved to permit “focused diversity”. It is likely that public dominant TCR, if selected for best fit, can rapidly recognize their antigen, while the highly private diverse side of the repertoire could be useful if the antigen mutates. The structure of both the TCR alpha and beta chain appear to play a role in interaction with the peptide/MHC complex to differing extents depending on the epitope. For instance, for many epitopes, including IAV-M1, the CDR3β plays the dominant role while for others, like EBV-BM, both chains contribute equally (3–6). The TCRα interaction often occurs with CDR1 or CDR2 rather than the CDR3. However, in two recent publications we have shown that CDR3α can play a critical role in selection of the TCR repertoire to the EBV-BR epitope due to structural constraints (7, 8). We have also shown that EBV-BM and EBV-BR repertoires are even more diverse and highly dynamic during an inflammatory response, acute infectious mononucleosis (AIM) (10,000 unique clonotypes/epitope/donor), than in healthy seropositive donors (1,000 unique clonotypes/epitope/donor). However, only 10% of the unique clonotypes present during AIM persist into memory, while the other 90% are replaced in 6 months with a completely new repertoire (7). It is important that we study and better understand epitope-specific TCR repertoire organization and how it evolves particularly with increasing age. As individuals age virus-specific immunity appears to wane.

Generally, TCRβ repertoire has been more extensively studied than TCRα, largely because techniques to study it, both antibody and sequencing, were easier to develop than for TCRα or alpha chain. However, it has become clear that TRAV gene segments can play an equally important role as TRBV in selection of antigen-specific repertoires as seen in EBV-BR specific TCR repertoires in patients with AIM (7, 8). As IAV-M1, EBV-BM, and EBV-BR TRBV repertoires are relatively well-documented and well-studied, many public TRBVs have been identified. BV19 has been identified as the most dominant BV family used in response to IAV-M1 (3); BV20, BV2, BV14, and BV29 in response to EBV-BM (7, 9–12) with anyone individual donor usually using one or two of these dominantly. Despite, the immunodominance of EBV-BR, it’s TCR repertoire is under-studied until recently. EBV-BR is unique in its ability to use multiple different TRBV families with an average of 4-5 different ones dominating in any one donor and often pairing with the public TRAV8.1 (7–9).

In addition, our lab has worked extensively to describe the concept of TCR cross-reactivity and explore changes to TCR repertoire in mouse models (1, 13–15) using viruses such as vaccinia virus (VV) (16, 17), lymphocytic choriomeningitis (LCMV) (18), IAV (19), CMV (20) and Pichinde virus (PV) (21, 22) that model chronic/persistent and acute viral infections in humans. As the results of these studies revealed an intricate network of TCR cross-reactivity between these viruses that cause acute and persistent viruses, our lab naturally pursued an examination of TCR cross-reactivity in humans. Two of the most common viruses that result in acute and persistent infections are IAV and EBV, respectively.

Our research is among the first to directly demonstrate that TCR repertoire determines severity of disease in humans (23, 24). In our studies using our well characterized AIM cohort we have documented expansions of EBV-specific and cross-reactive CD8 T cells in primary EBV infection and mapped a network of cross-reactive CD8 T cell responses between EBV and another common human virus, IAV (25, 26). AIM varies in severity from a mild transient flu-like illness to a prolonged and severe syndrome. In 32 young adults with AIM, we found that disease severity directly correlated with the frequencies of IAV-M1+ and IAV-M1+EBV-BM+ tetramer+ CD8 T cells (and weakly with EBV-BM) (23). Moreover, memory IAV-M1-specific CD8 T cell frequencies > 0.36% (direct ex vivo tetramer staining) were associated with a 5-fold greater risk of severe AIM. IAV-M1 tetramer+ cells were functionally cross-reactive, proliferating to and producing cytokines to EBV-BM. Cross-reactive IAV-M1-specific CD8 T cells associated with severe AIM had a distinct TRBV usage that correlated with disease severity (23).

However, this cross-reactivity between IAV-M1 and EBV-BM may also protect against EBV infection depending on the TCR repertoire. By early adulthood, 95% of the population has been infected with EBV, but 5% of individuals remain seronegative even when they should have been exposed and yet appear to resist infection (27). We have identified 5 rare individuals, who were EBV seronegative, who had elevated IAV-M1 tetramer+ CD8 T cell frequencies *ex vivo (* 24). EBV-BM or BR-stimulated cultures from these donors exhibited high frequencies of cross-reactive IAV-M1 tetramer+ cells. These cultures produced IFNγ to EBV epitopes and lysed EBV-infected targets, suggesting that these individuals may indeed be protected from infection. They had highly unique oligoclonal IAV-M1-specific TCR repertoires that differed from young EBV seronegative donors (24). Altogether, these two studies link heterologous immunity *via* cross-reactive CD8 T cells to CD8 TCR repertoire selection, function, and disease outcome in a common and important human infection.

To help us better understand how TCR repertoire may influence disease outcome recent studies have shown that there is now enough data available from MHC/peptide structures and antigen-specific TCR sequencing databases to develop novel algorithms that could assist in using the TCRa and TCRb repertoire sequences to track epitope-specific repertoires (6, 28). Paul Thomas and colleagues (6) developed an algorithm examining single cell TCR sequences, TCR distance measure, TCRdist, that enabled visualization of the epitope-specific repertoires through clustering and dimensionality reduction. To calculate TCRdist scores between 2 TCRs, each TCR is first mapped to the amino acid sequences using a similarity-weighted Hamming distance, with a gap penalty introduced to capture variation in length and a higher weight given to the CDR3 loop. This algorithm can help identify for any antigen-specific response the preferential usage of TCR BV/BJ/AV/AJ and their preferential pairings. This algorithm also could define the preferential usage of particular amino acids in certain positions of the CDR3 as compared to other TCR in the antigen-specific population (motif 1) and as compared to a naïve TCR repertoire (motif 2). This information can be used to identify which features of the TCR are public and important for interaction with that ligand. Once one is able to identify the distance between TCRs one can potentially predict how they cluster based on similar traits and potentially which antigen they might recognize and their potential to recognize two antigens and be cross-reactive. Mark Davis and colleagues (29) used a similar approach called GLIPH to identify public features of TCR that were activated by *M. tuberculosis* stimulation in infected patients. They constructed the TCR and inserted them into Jurkat cells and screened a plasmid library of *M. tuberculosis* peptides to identify their ligands. These technologies would be particularly useful for defining TCRs that recognize potentially cross-reactive low affinity and hard to identify ligands such as in autoimmune diseases, or cancer.

Despite the development of robust EBV-specific humoral (30) and cell-mediated immunity (31–34), EBV establishes persistence *via* latent infection of memory B cells (35). In healthy people, EBV is known to continuously go into lytic cycle and the immunosuppression of an acute IAV infection may further increase the rate of reactivation. Thus, we would predict that being infected with two viruses at the same time would greatly enhance selection of CD8 T cells that are cross-reactive during acute IAV infection. We have evidence that not only IAV-M1, but also EBV-BM and EBV-BR tetramer frequencies increase during acute asymptomatic IAV with changes in their TCR repertoire (36). Here, we dissected IAV-M1, EBV-BM and EBV-BR TCRαβ repertoires in the two age groups, young and older donors, all persistently infected with EBV and previously exposed to IAV. We show with the assistance of TCR dist analyses of not only TRBV, but the under studied TRAV high throughput sequence and single cell data, that there are definable changes in epitope-specific TCR repertoires to these two ubiquitous viruses with increasing age influenced by TCR cross-reactivity.

## Materials and methods

### Study population

Our studies include young adults and older adults that are healthy, HLA-A2.01+, IAV-immune and EBV seropositive. EBV serology was confirmed by the presence of viral capsid antigen (VCA) IgG specific antibodies in addition to staining with EBV-specific tetramers. IAV immunity was confirmed by staining with IAV-specific tetramers. The young adults (Y) (18-21 years old) in this study were a part of an EBV Sero-surveillance cohort developed by Drs. Liisa Selin and Katherine Luzuriaga at University of Massachusetts Amherst (UMA). These donors were followed from freshman year to senior year, during which they donated blood once a semester. Older donors (O) (>60 years old) were volunteers acquired at University of Massachusetts Medical School (UMMS). Volunteers were allowed to donate up to 150ml blood in 3 months, in accordance with our IRB. All participants in this study were required to sign a consent form. This study was approved by the Institutional Review Board (IRB) committee at University of Massachusetts Medical School, Worcester, Massachusetts.

### HLA-typing

Monoclonal antibodies specific to HLA-A2.01(clone BB7.2, Biolegend, San Diego, CA, HLA-B8.01 (clone BB7.1, Santa Cruz Biotechnology, Dallas, TX), and HLA-B7.01 (clone 8.L.215 Biotin, Abcam, Cambridge, MA) were added to 100ul of whole blood and stained for 30 minutes in the dark at room temperature. Cells were washed with 1ml of Hank’s Balanced Salt Solution (HBSS) (Gibco, Grand Island, NY) and spun at 1330rpm for 4mins, 25°C. The cells were incubated in the dark for 30 minutes, then washed with 1ml of HBSS. PE Streptavidin (Biolegend, San Diego, CA) was added to the cells and incubated for 30 minutes and washed. To lyse red blood cells, 2ml of 1X BD FACS Lysing Solution (Becton Dickinson, Waltham, MA) were added for 10 minutes. Cells were washed once with HBSS and spun. Cells were resuspended in 300ul FACS buffer (500ml HBSS, 2% Fetal Calf Serum) and analyzed on the LSRII (Becton Dickinson, Waltham, MA).

### PBMC isolation

Fresh whole blood was mixed with Hank’s Balanced Salt Solution (Gibco, Grand Island, NY) at 1:2 ration and half of this mixture was layered over 15mls of Ficoll-Paque Plus (GE Healthcare Bio-Sciences, Pittsburgh, PA) (23). Layered cells were placed in a centrifuge and spun at 1800rpm for 40 minutes with no brake at 25°C. PBMC from the buffy coat were collected and washed twice with 20ml HBSS.

### CD8 T cell isolation

Counted and re-suspended PBMC in 20μl of anti-CD8 micro-beads (Miltenyi Biotech, Auburn, CA) and 80μl of MACS buffer [4°C Phosphate-buffered saline, 2.5g of Bovine Serum Albumin (Sigma-Aldrich, St.Louis, MO)], 2ml 0.5M EDTA [pH 8.0 (Invitrogen, Grand Island, NY)] degassed with sterile mesh filter] per 10^7^ cells based on Miltenyi MACS system protocol (23). PBMC and anti-CD8+T micro-beads mixture were incubated in the dark at 4°C for 15 minutes. Mixture was washed with 20ml of MACS buffer. Miltenyi Biotech MACS system was used to isolate CD8+T cells.

### CD8+ T cell short-term culture

HLA A*0201 specific transporter associated with antigen transport (TAP)-deficient T2A2 cells, which express low amounts of MHC Class-I protein on their surface, were used as antigen presenting cells (3, 23, 24, 26). Cells were plated at 4 x 10^6^ T2A2 (ATCC #CRL-1992) cells per 3ml of T2A2 media (500ml RPMI, 10% Fetal Calf Serum, 1% HEPES, 1% Penicillin-Streptomycin, 1% L-Glutamine) for 3 hours at 37°C with 1μM of peptide (final concentration= 1mM). T2A2 cells were irradiated with 3000 RAD and washed to remove unbound peptide. T2A2 cells re-suspended in T cell media [AIM-V (Gibco, Grand Island, NY) supplemented with 14% human AB serum [(Interstate Blood Bank INC, Memphis, TN), 16% MLA-144 supernatant (Rabin et al, 1981), 10 U/ml human rIL-2 (Becton Dickinson, Waltham, MA), 1% L-Glutamine (Gibco, Grand Island, NY), 0.5% β-mercaptoethanol (Sigma-Aldrich, St.Louis, MO), 1% HEPES (Hyclone, Logan, UT)]. Plated 1 x 10^6^ of CD8+ T cells with 2 x10^5^ T2A2 cells loaded with a single peptide in a 4ml total volume of T cell media into a 12 well plate were cultured for 3 weeks.

### Method to study crossreactivity

In these studies we assessed both types of cross-reactive CD8 T cells, single tetramer+ and double tetramer+ from IAV-M1 peptide stimulated short term cultures (23, 26). In order to examine single tetramer+ cross-reactive CD8 T cells we sorted EBV-BM (M1BM) or EBV-BR (M1BR) tetramer+ cells from IAV-M1 stimulated short term cultures for TCR high throughput sequencing. We also sorted M1+BR+ double tetramer+ cells from the IAV-M1 stimulated short-term cultures of two young donors who had this population. We used the same methodologies as previously (23, 26), where we did all of the same controls in our culture system, stimulating short term IAV-M1, EBV-BM, EBV-BR, tyrosinase and CMV-pp65 cell lines on each magnet sorted CD8 T cell population of each donor. This is a useful technique to study lower affinity functional cross-reactivity as, we observe crossreactive cells, for instance EBV-BR tetramer binding cells growing in IAV-M1 stimulated cultures only and not growing in any of the other cultures which act as controls. With this method we study both functional single tetramer binding crossreactivity and double tetramer staining crossreactivity. This culturing technique allows us to circumvent issues with tetramers blocking the binding of the other tetramer during crossreactive responses due to differing affinities, as we have previously described can be a significant problem particularly ex vivo (23, 26).

### Peptides

CD8+ T cells were stimulated with IAV-specific and EBV-specific peptides that were synthesized to >90% purity (21^st^ Century Biochemical, Marlborough, MA). The following lytic EBV peptides were used: EBV-BMLF1**_280-288_** (GLCTLVAML) and EBV-BRLF1**_109-117_** (YVLDHLIVV). For IAV-M1 specific responses, CD8+ T cells were stimulated with IAV-M1**_58-66_** (GILGFVFTL). T2-A2 cells were pulsed with peptides and used at concentration of 0.1 mg/ml. Peptides used for intracellular assays were used at a 1mg/ml concentration in Dimethyl sulfoxide (Sigma-Aldrich, St. Louis, MO).

### Tetramers and dextramers

IAV-M1 tetramer, EBV-BMLF1 tetramer, EBV-BRLF1 tetramer were provided by in-house tetramer core facility and NIH Tetramer Core Facility (Atlanta, GA). These tetramers including IAV-M1 dextramer (Immudex, Copenhagen, Denmark) were assembled and conjugated to either allophycocyanin (APC) or phycoerythrin (PE) or brilliant violet (BV) 421. Tyrosinase (in-house tetramer core facility and NIH Tetramer Core Facility, Atlanta, GA) and CMV_pp65_ (in-house tetramer core facility and NIH Tetramer Core Facility, Atlanta, GA) were used as negative controls for all experiments.

### Extracellular staining

3 x 10^5^ freshly isolated or cultured CD8+ T cells were placed into a 96 well plate. Cells were stained with tetramers and dextramers for 30 minutes at room temperature (RT). Cells were washed twice with FACS buffer (500ml Hank’s Balanced Salt Solution with 2% Fetal Calf Serum). Cells were fixed using 100μl of Cytofix (Becton Dickinson Biosciences, San Jose, CA) for 5 mins in the dark at RT. The cells were washed, spun at 1330rpm for 4mins, 25°C and re-suspended in FACS buffer and prepared for flow cytometry.

### Intracellular staining

Cell were prepared at 1 x 10^6^ cells in 200μl of T cell media with Golgi-Stop, Golgi-Plug (Becton Dickinson Biosciences, San Jose, CA), which allowed the accumulation of cytokines in the Golgi-complex, and anti-CD107a/b antibodies (mAb eBioH4A3, eBiosciences, San Diego, CA). Cultured cells were incubated for 5 hours at 37°C in the presence of 5μM of the same peptide used for 3-week stimulation. Cells were washed twice in FACS Buffer and spun down to remove unbound peptides and antibodies, the cells were washed twice using FACS buffer. Cells used in the cell surface stain with dextramers and tetramers were incubated for 30 minutes at RT. Cells were washed twice and fixed. Cells were then permeabilized with Cytofix/Cytoperm (Becton Dickinson Biosciences, San Jose, CA) for 20 minutes at RT. Cells were washed twice using FACS buffer. The following antibodies were used to detect production of cytokines: anti-IFN-γ (0.2μg clone B27, Becton Dickinson, San Jose, CA), anti-MIP-1β (0.2μg clone D21-1351, Becton Dickinson, San Jose, CA), and anti-TNF-α (mAb11, eBiosciences, San Diego, CA) for 30 minutes at RT. Cells were washed twice and fixed using Cytofix (Becton Dickinson Biosciences, San Jose, CA) for 20 minutes. Cells were washed and spun at 1330rpm for 4mins, 25°C then re-suspended in FACS buffer for flow cytometry.

### CD8 T cell sorting

Freshly isolated or cultured CD8+ T cells stained with tetramers and dextramers were collected into a 1.5ml FACS tubes with 400μl of FACS buffer. In order to examine whether and how cross-reactivity might influence or change TCR repertoire with increasing age we also assessed both types of cross-reactive CD8 T cells, single tetramer positive and double tetramer from IAV-M1 peptide short term cultures (23, 26). In order to examine single tetramer+ cross-reactive CD8 T cells we sorted EBV-BM (M1BM) or EBV-BR (M1BR) tetramer+ cells from IAV-M1 stimulated short term cultures for TCR high throughput sequencing. These were present in both young and older donors. We also sorted M1+BR+ double tetramer+ cells from the IAV-M1 stimulated short-term cultures of two young donors who had this population. Cells were sorted at the University of Massachusetts Medical School FACS Core Facility in the Biosafety Level 3 (BSL-3) suite (UMASS Medical School, Worcester, MA), using a BSL-3 BD FACS Aria Cell Sorter.

### TcR V beta repertoire staining

The TcR V beta repertoire kit contained antibodies to 24 V beta families (Beckman Coulter, Fullerton, CA). Cells were stained with these antibodies and tetramers or dextramers for 20 minutes at RT to determine the V beta repertoire of antigen specific cells. The cells were washed twice in FACS Buffer and spun. Cell were resuspended in FACS buffer and analyzed using flow cytometry.

### TCR repertoire high throughput sequencing

Tetramer-positive cells were sorted and then RNA isolated. Following preparation of a cDNA library, samples were sent to Adaptive Biotechnologies, Seattle, WA). TCRα and TCRβ repertoires data were analyzed using ImmunoSEQ Analyzer version 2.0, available online through Adaptive Biotechnologies. **Supplemental Table S4** summarizes the TCR sequencing characteristics of each sorted population sequenced. The detailed TCR sequencing data can be accessed *via* in the Adaptive Biotechnologies database at Email: gil-review@adaptivebiotech.com; Password: gil2022review.

### Single cell PCR

Tetramer+ CD8 T cells were single cell sorted on FACS Aria (Becton Dickinson, San Jose, CA) into 96-well plates and prepared for total RNA isolation (Qiagen, Hilden, Germany). After reverse transcription into cDNA [SuperScript VILO cDNA synthesis kit (Invitrogen)] the PCR was performed following the protocol previously described (8). CDR3 amplicons were purified (ExoSAP-IT) and sequenced with primers that recognized constant regions of TRAC and TRBC. Sanger DNA sequencing was performed by Genewiz (Cambridge, MA).

Statistics: Pearson correlation and 2 way-ANOVA multi-variant analysis with correction for multiple comparisons was used to analyze data. TCRdist was used to analyze the paired single cell data [analysis method from Dash et al. (6); Kamga et al. (8)]. A modified version of TCRdist was used to analyze the high throughput TRAV or TRBV repertoire data, which is available on the following website: https://github.com/thecodingdoc/tcrdistScripts.

## Results

### Characteristics of patient populations and CD8 T cell populations

For these TCR repertoires studies, we recruited and enrolled healthy, IAV-and EBV-immune, HLA-A2.01+ donors. We used 2 age groups defined as young, 18-22 years old, and older, >60 years old (**Supplemental Tables S1A, B**). For the TCR high throughput sequencing studies, the average age of the 4 young EBV sero-positive donors was 19±1 years old, and for the 5 older EBV sero-positive donors was 71±4. We studied the two extremes of age as our earlier studies (36) indicated significant changes in these particular virus-specific TCR repertoires. Also, our understanding of epitope-specific TCR repertoires in both young and older donors in comparison to middle-aged donors is still limited.

We have previously determined the CD8 memory T cell frequencies and TRBV repertoires by mAb staining to IAV-M1, EBV-BM and EBV-BR epitope-specific responses *ex vivo* in these same individuals in a cross-sectional study (36). Here, we will examine in more detail the differences in both TRBV and TRAV usage in these two age groups by high throughput sequencing and single cell sequencing of tetramer positive cells. For these rather extensive studies we need to use large numbers of cells so we did short term culture with peptide stimulation using a technique that we have published on extensively (9, 23, 24) (**Supplemental Figures S1A-D**). In previous studies (3, 9) and this manuscript, we showed that the same BV families are used before and after stimulation with peptide and there is a high degree of correlation but there are some shifts in the relative proportions that do not rule out differential expansion altogether (i.e. TRBV4/5/6 for IAV-M1, TRBV-3 for EBV-BRLF1 in the current data). In particular, we will focus on studying not only virus-specific differences, but also cross-reactive CD8 TCR repertoires to assess if cross-reactivity may play a role in the co-evolution of virus-specific TCR repertoires with increasing age (**Supplemental Figures S1A, B**).

### TRBV family usage and diversity as measured by monoclonal antibody staining for EBV epitope-specific responses differs between young and older donors

**Figure 1:**
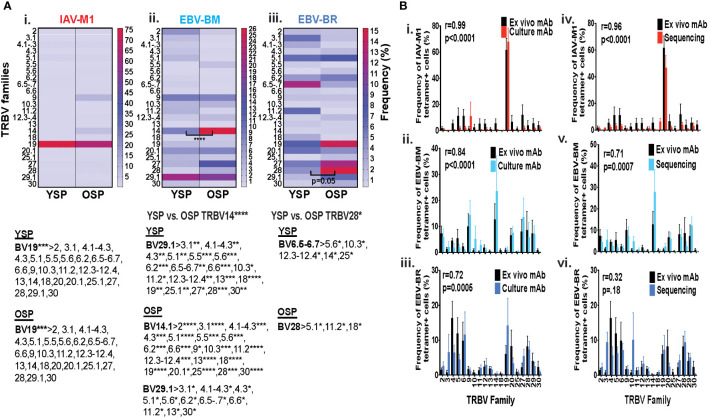
TRBV usage **(A)** as measured by mAb staining for EBV epitope-specific responses differs between young (Y) and older (O) donors. Following short-term culture with either IAV-M1, EBV-BM or EBV-BR peptide, cognate (same specificity as the stimulating peptide) tetramer+ cells in each culture were stained with TRBV8 mAbs (Y n=12-13, O n=7-9). **(A)** Heatmap analysis shows that TRBV usage differed between Y and O donors in EBV-specific responses either when frequency was directly compared between the groups or if the hierarchy of TRBV usage within the group was examined. A single TRBV family, BV19, dominated IAV-M1-specific responses in both Y and O donors. The dominant BV usage for each specificity is shown below each heatmap. **(B)** Strong correlations in TRBV usage between short-term cultured and *ex vivo* antigen-specific responses. TRBV repertoire as assessed by TRBV monoclonal antibody (mAbs) staining of *ex vivo* tetramer+ CD8 T cells were compared to those in short-term culture in the same donors as assessed by BV mAb staining (i-iii) or TCR high throughput sequencing (iv-vi) (Y n=4, O n=4-5). Multi-variant 2-way ANOVA with adjusted p-value, *p < 0.05, **p < 0.01, ***p < 0.001, ****p < 0.0001 **(A, B)**. Pearson’s correlation coefficient (r), r and p values indicated on graph.

Initially, using tetramer and TRBV monoclonal antibody mAb co-staining of epitope-specific cells from short term culture on a larger number of donors (**Supplemental Table S1**) we observed that there were significant differences in the pattern of TRBV usage of EBV-BM and EBV-BR specific responses between older and young donors (EBV-BM older BV14 3-fold > young; EBV-BR older BV28 3.5-fold > young) (**Figure 1A**). There also was a significant change in preferential hierarchy of TRBV usage for the two EBV-specific epitopes within each donor group (**Figure 1A**ii-iii**).** In EBV-BM responses young preferentially used BV29.1, while older donors used BV29.1 and BV14.1. In EBV-BR responses, the young preferentially used BV6.5, while older used BV28. Interestingly, for IAV-M1 responses, TRBV19 was highly dominant in both groups as has been previously reported for older and middle aged donors (6, 37, 38). These changes in TRBV usage, particularly in the EBV-specific responses are consistent with our *ex vivo* findings (36) and are highly suggestive that TRBV repertoire does evolve and change with increasing age.

### Strong correlations in TRBV usage between short-term cultured and *ex vivo* antigen-specific responses

In order to determine if short-term culture would alter the *ex vivo* antigen-specific TCR repertoire we compared tetramer+ CD8 T cell repertoires of the short-term cultured cells either by mAb staining or high throughput sequencing to *ex vivo* mAb staining. The TRBV repertoire frequencies in short term culture as measured by mAb staining or TCR high throughput sequencing when using the mean value for the same young and older donors (**Supplemental Table S1A**), where data was available using both methods, directly correlated with the *ex vivo* mAb staining results for IAV-M1, EBV-BM and EBV-BR epitope-specific responses (**Figure 1B**i-iii). It should be noted that we did observed some global functional differences in the young and older cultured CD8 T cells (**Supplemental Figures S1C, D**), but this did not affect their TCR repertoires (**Figure 1B**). These results suggest that our short-term culture method does not significantly alter epitope-specific TCR repertoires.

### Specific features of the CDR3 dominate in IAV-M1 and EBV-cognate and cross-reactive TCR repertoires with increasing age

There are certain general CDR3 features that have been reported to dominate in antigen-specific CD8 T cell responses, which include increased usage of amino acids with convergent recombination (RAA) (increased usage of amino acids that have multiple ways of being derived) (39–41), increased N nucleotide additions to the VDJ joining region (NNA), and increased usage of multiple glycines or glycine runs (GGG). Multiple glycines, in particular, have been associated with increased flexibility and cross-reactivity (42, 43). There is some evidence, that there is a greater ease of generation of TCRs that use CDR3 with convergent recombination and shorter CDR3 (less N nucleotide additions) (44, 45). We were interested in determining if with increasing age there was a greater selection of TCR that have these features in both the virus-specific (cognate) and cross-reactive repertoires. In order to obtain more detailed information about TCR repertoire changes in TRBV, but also in TRAV required TCR high-throughput sequencing of tetramer-sorted epitope-specific and cross-reactive populations.

**Figure 2:**
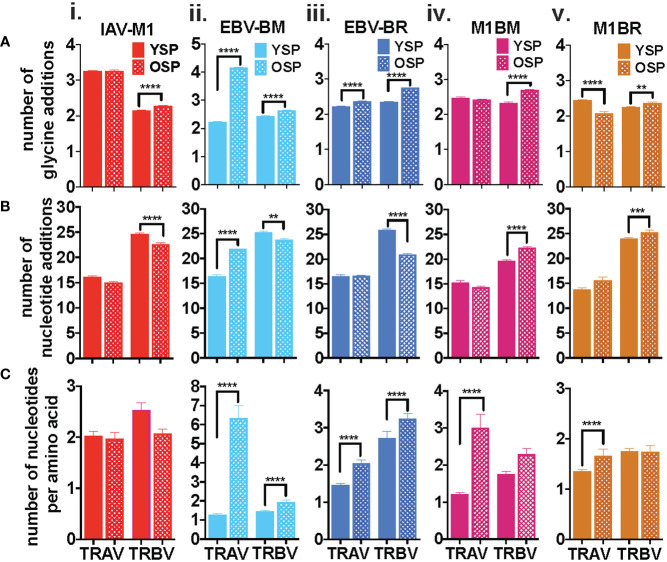
Specific features of the CDR3 dominate in IAV-M1 and EBV-cognate and cross-reactive TCR repertoires with increasing age. IAV-M1- and EBV-BM and EBV-BR- specific (cognate) and cross-reactive short-term cultured cells from younger and older donors were tetramer-sorted for high throughput sequencing (Y n=4, O n=4-5). M1BM or M1BR are cross-reactive EBV-BM or EBV-BR single tetramer+ cells sorted from IAV-M1 stimulated short term cultures. Significant differences were found in the number of glycines (**A**i-v, number of nucleotide additions (**B**i-v), and number of nucleotides per amino acids (**C**i-v) between Y and O and between the epitope specific and cross-reactive responses (**Figure S1**) for TRAV and TRBV. Multi-variant 2-way ANOVA with adjusted p-value, **p < 0.01, ***p < 0.001, ****p < 0.0001.

The IAV-M1, EBV-BM and EBV-BR cognate and cross-reactive TRBV and TRAV repertoires differed significantly between the older and young donors in use of RAA, NNA and GGG (**Figures 2A–C**) as summarized in **Supplemental Table S2**. As there were many significant differences between older and young, we will highlight some of the most important ones. The most consistent change in CDR3 features between the groups, was an increased retention in older of GGG in the TRBV of all five epitope-specific repertoires, cognate IAV-M1, EBV-BM, EBV-BR, and cross-reactive M1BR and M1BM (**Figure 2A**i-v), as well as, three of the TRAV epitope-specific repertoires, EBV-BM, EBV-BR and M1BR (**Figure 2A**ii,iii,v). This suggests a greater retention of potentially more flexible TRAV and TRBV chains or TCRs that could have double usage as cross-reactive TCR with increasing age.

Older donors showed significantly less NNA in TRBV cognate IAV-M1, EBV-BM and EBV-BR specific responses than young (**Figure 2B**i-v), which suggests a retention of TCR that are potentially easier to make. However, the older cross-reactive responses, M1BM and M1BR, had more NNA than young suggesting that they are retaining longer CDR3 that may enhance their cross-reactivity. Older donors also showed a significantly increased usage of RAA in both TRAV and TRBV for EBV-BM and EBV-BR-specific responses (**Figure 2C**i-v) suggesting that TCRs easier to generate are retained with increasing age.

**Figure 3:**
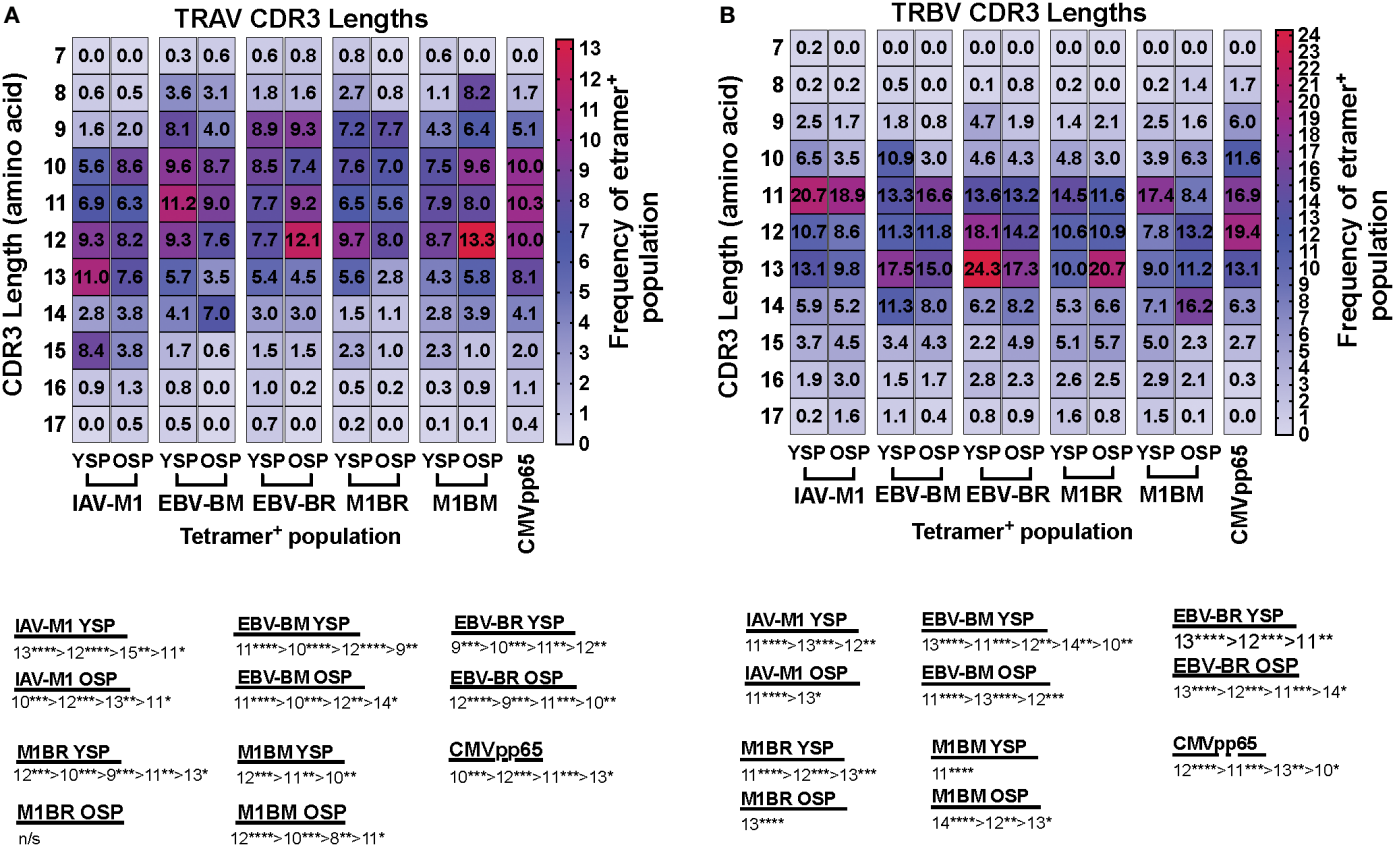
TRAV and TRBV CDR3 lengths of IAV-M1 and EBV epitope specific responses differ between young and older donors. TRAV and TRBV CDR3 lengths (amino acids) were determined for IAV-M1, EBV-BM, and EBV-BR cognate and cross-reactive short-term cultured CD8 T cells that were tetramer-sorted and sequenced (Y n=4, O n=4-5). M1BM or M1BR are cross-reactive EBV-BM or EBV-BR single tetramer+ cells sorted from IAV-M1 stimulated short term cultures. CMVpp65 epitope specific responses were used as a control, which included young and middle-aged donors (n=3). Heatmap analyses of preferential TRAV CDR3 length usage shows different preferential hierarchies between different epitope-specific responses and for the same epitope between Y and O in TRAV **(A)** and TRBV **(B)**. Below heatmap is the hierarchy of the dominant CDR3 lengths used by the indicated response. Multi-variant 2-way ANOVA with adjusted p-value, *p < 0.05, **p < 0.01, ***p < 0.001, ****p < 0.0001.

We also noted some differences in the overall pattern of TRAV (**Figure 3A**) and TRBV (**Figure 3B**) CDR3 lengths in the IAV-M1 and EBV epitope-specific responses between young and older donors. In the TRAV repertoires, the older used relatively similar CDR3 lengths to the young donors, except they used a shorter CDR3 (older, 10-mer vs younger, 11-mer) in the IAV-M1 response and a longer CDR3 (older: 12-mer vs young: 9-mer) in the EBV-BR. Overall, the cross-reactive TRAV and TRBV of the cross-reactive M1BR and M1BM responses used longer CDR3 than their corresponding cognate response and the older had even longer CDR3 than young in the TRBV (M1BR: older, 13-mer vs young, 11-mer; M1BM: older, 14-mer vs young, 11-mer).

Overall, these results would suggest that with increasing age there is a preferential selection or retention of TCR that have CDR3 features that increase their ease of generation and cross-reactive potential.

### TRAV, TRAJ, TRBV and TRBJ family usage in IAV and EBV-specific and cross-reactive responses differ between young and older

**Figure 4:**
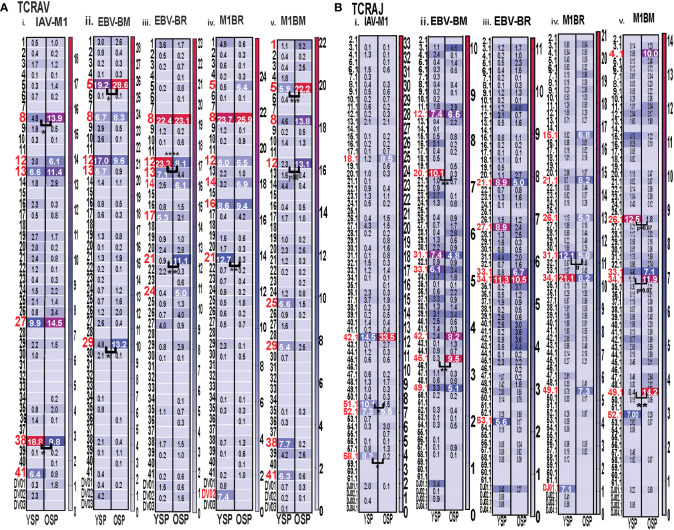
Significant differences in TRAV and AJ family usage in IAV and EBV-specific and cross-reactive responses between young (Y) and older (O) donors. TRAV and AJ families determined for IAV-M1, EBV-BM, and EBV-BR cognate and cross-reactive responses in short-term cultured CD8 T cells that were tetramer-sorted and sequenced (Y n=4, O n=4-5). M1BM or M1BR are cross-reactive EBV-BM or EBV-BR single tetramer+ cells sorted from IAV-M1 stimulated short term cultures. **(A)** Heatmap analyses of TRAV **(A)** and AJ **(B)** family usage, showed significant differences in epitope-specific responses between Y and O. Multi-variant 2-way ANOVA with adjusted p-value, *p < 0.05, **p < 0.01, ***p < 0.001, ****p < 0.0001. (Also see **Supplemental Table S3** for statistical analyses).

With the use of TCR high-throughput sequencing and heatmap display we were able to show that there were changes not only in TRBV but also TRAV family usage as well as J family usage with increasing age for all three epitope specific responses. For both age groups, all of the cognate responses predominantly used the classic public TRAV that have been previously reported (3, 7, 9), (IAV-M1: AV27, AV38; EBV-BM: AV5, AV8, AV12; EBV-BR: AV8, AV12) (**Figure 4A**i-iii). The cross-reactive M1BR response used both AV8, AV12 but also AV5 (public for EBV-BM), AV16, AV14 and AV21 (**Figure 4A**iv). The cross-reactive M1BM response used AV5, AV8, AV12 but also, AV1, AV25, AV29, AV38 (public for IAV-M1) and AV41 (**Figure 4A**iv). There were, however, significant differences in AV family usage between older and young in both cognate (IAV-M1: AV8, older>young; AV38, older<young; EBV-BM: AV5, older>young, AV29, older>young; EBV-BR: AV12, older<young; AV21, older>young) and cross-reactive responses (M1BR; AV21, older<young; M1BM: AV5, older>young; AV12, older>young) (**Supplemental Table S2**; statistical analyses shown in **Supplemental Table S3**).

For both age groups, all of the cognate responses predominantly used the classic public TRAJ families that have been previously reported (3, 7, 9), (IAV-M1: AJ42, AJ52; EBV-BM: AJ31, AJ12; EBV-BR: AJ34, A21) (**Figure 4B**i-ii). The cross-reactive M1BR response used both AJ34, AJ21 but also AJ31 (public for EBV-BM), AJ27, AJ26, AJ49 and DJ01.1 family (**Figure 4B**iv). The cross-reactive M1BM response did not use AJ31 but instead used AJ34 (public for EBV-BR), AJ26, AJ33, AJ49, and AJ52 (public for IAV-M1) (**Figure 4B**v). There were, however, significant differences in AJ family usage between older and young in both cognate (IAV-M1: AJ51, older<young; AJ58, older<young; EBV-BM: AJ45, older>young, AJ29, older<young) and cross-reactive responses (M1BR; AJ31, older<young; M1BM: AJ26, older<young; AJ34, older>young; AJ49, older>young) (summarized **Supplemental Table S2**, statistical analyses shown in **Supplemental Table S3**).

**Figure 5:**
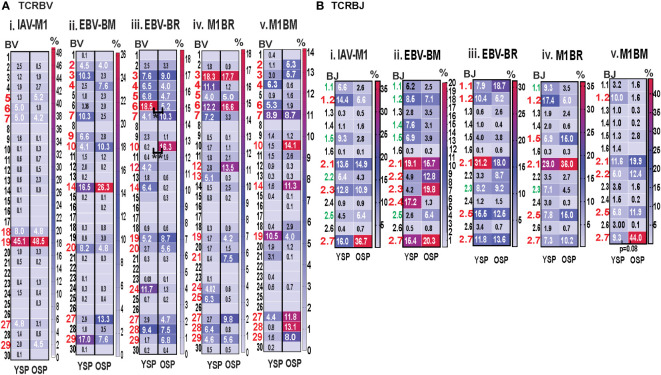
Significant differences in TRBV and BJ family usage in IAV and EBV-specific and cross-reactive responses between young (Y) and older (O) donors. TRBV and BJ families determined for IAV-M1, EBV-BM, and EBV-BR cognate and cross-reactive responses in short-term cultured CD8 T cells that were tetramer-sorted and sequenced (Y n=4, O n=4-5). M1BM or M1BR are cross-reactive EBV-BM or EBV-BR single tetramer+ cells sorted from IAV-M1 stimulated short term cultures. **(A)** Heatmap analyses of TRBV **(A)** and BJ **(B)** family usage, showed significant differences in epitope-specific responses between Y and O. Multi-variant 2-way ANOVA with adjusted p-value, *p < 0.05, **p < 0.01. (Also see **Supplemental Table S3** for statistical analyses).

For both age groups, all of the cognate responses predominantly used the classic public TRBV families that have been previously reported (3, 7, 9), (IAV-M1: BV19; EBV-BM: 10 different BV including BV14, BV29, BV20, BV2, BV9, BV10; EBV-BR: 14 different BV including BV6, BV3, BV4, BV5, BV19, BV, 27, BV28) (**Figure 5A**i-iii). The cross-reactive M1BR response also used 14 different BV with a greater usage of BV3 than in cognate EBV-BR (**Figure 5A**iv). The cross-reactive M1BM response used 11 different BV including a greater usage of BV19 (public for IAV-M1) than in cognate EBV-BM responses. (**Figure 5A**iv). Although the overall hierarchy and pattern of BV family usage appeared to differ between older and young for each epitope, there were few significant differences in BV family usage between older and young in both cognate (EBV-BR: BV6, older<young; BV10, older>young) and cross-reactive responses. There are only 13 different TRBJ families and there was dominant usage of BJ2.1, and BJ2.7 by all cognate and cross-reactive responses with no major differences between older and young donors. (**Supplemental Table S2**, statistical analyses shown in **Supplemental Table S3**).

Since we are interested in determining whether TCR cross-reactivity could play a role in the changes in TCR repertoire with increasing age to these three epitopes it is noteworthy that AV8 and AV12 family are dominantly used by all three epitope-specific responses, as well as, both cross-reactive responses. Young donors used TRAV21 family in their M1BR response, while older used TRAV21 in their cognate BR response. Older had a dominant usage of TRAV5 in both cognate and cross-reactive BM responses, which may suggest cross-reactivity is playing a role in the dominant selection of this TRAV in EBV-BM responses. This public EBV-BR TRAV8 family usage was significantly increased in the older IAV-M1 response as compared to young. TRAV12 was common in EBV-BM, EBV-BR and significantly used more by older cross-reactive M1BM than in young donors. The dominant TRAJ family for M1BM and M1BR responses differed from their cognate counterparts in young and older suggesting that features of TRAJ may play a role in the specificity of TCR cross-reactivity. There is also a great deal of overlap between the dominant TRBV usage of the 3 cognate and 2 cross-reactive responses, including BV19, BV3, BV7, BV27, BV6 and BV29. These types of overlaps in AV, BV, BJ usage between epitope-specific responses greatly increases the chance that these TCR repertoires could contain cross-reactive TCR.

Overall, the TCRb high throughput sequencing data was consistent with the mAb staining data showed in unpublished manuscript before, in that there were fewer significant direct differences in TCRb usage than TCRa between young and older, although there were hierarchy differences. If cross-reactivity is driving the change in TCR repertoire with increasing age this may arise from the fact that there is a great deal of overlap in BV usage between these epitope-specific responses. These data could be interpreted to suggest that perhaps TRAV usage may play a greater role in evolution of the TCR repertoire and in determining specificity of TCR cross-reactivity.

### *TRAV* and *TRAJ* gene usage, pairing and CDR3 motifs of IAV-M1, EBV-BM and EBV-BR differ between young and older donors

**Figure 6:**
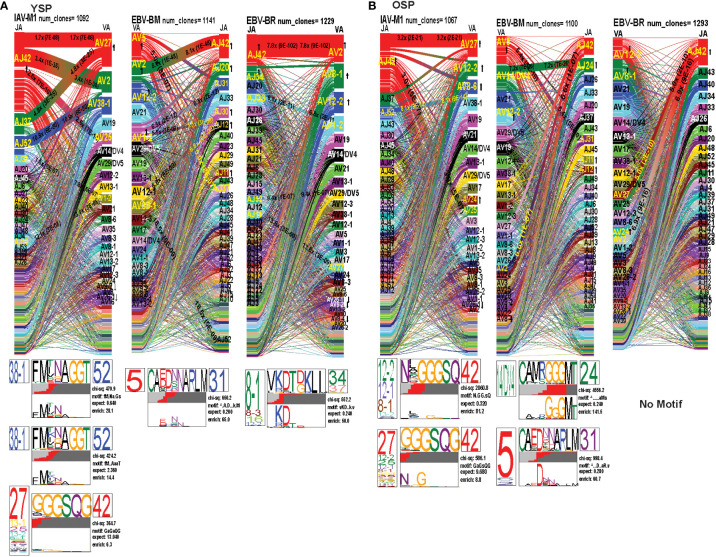
The TRAV and TRAJ gene pairing and CDR3 motifs for IAV-M1, EBV-BM and EBV-BR differ between young (Y) and older (O) donors. This was determined after TCR high throughput sequencing of tetramer-sorted CD8 T cells in Y and O donors (Y, n=4; O, n=4-5) using ribbon-plot analyses. Ribbon plots show patterns of TCR V-J pairings in TRAV in young **(A, B)** older donors (num_clones, indicates the number of clones analyzed). Genes are colored by frequency within the repertoire with red>green>blue>cyan>magenta>black. The arrows indicate significant fold increase usage of those V or J regions compared to naïve random repertoire suggesting antigen-driven expansion (no. of arrow heads are log_2_). Underneath each ribbon plot are the unique clearly defined CDR3 motifs of the TRAV repertoire of the indicated antigen-specificity. There can be multiple different CDR3 motifs for any one specificity. For each CDR3 motif, the upper motif 1 (labeled Mf1 in Y IAV-M1 as a representative) shows the amino acids which are enriched in comparison to the total tetramer+ population of that specificity; the lower motif 2 (labeled Mf2 in young IAV-M1 as a representative) shows the amino acids which are enriched compared to a naïve random CD8 T cell repertoire. Both indicate that the identified amino acids are important for an antigen peptide/MHC contact. Naive repertoires do not generate motifs as this requires the presence of clonal expansions. (analysis method from Dash et al. (6); Kamga et al. (8). The text within the bars joining particular AV and AJ regions indicate the fold increased usage of that pairing (and statistical significance) compared to a naïve random TCR repertoire. Bar in between the Mf1 and Mf2 depicts which part of the CDR3 is derived from the V (light grey), N (red), D (black) and J (dark gray) regions.

**Figure 7:**
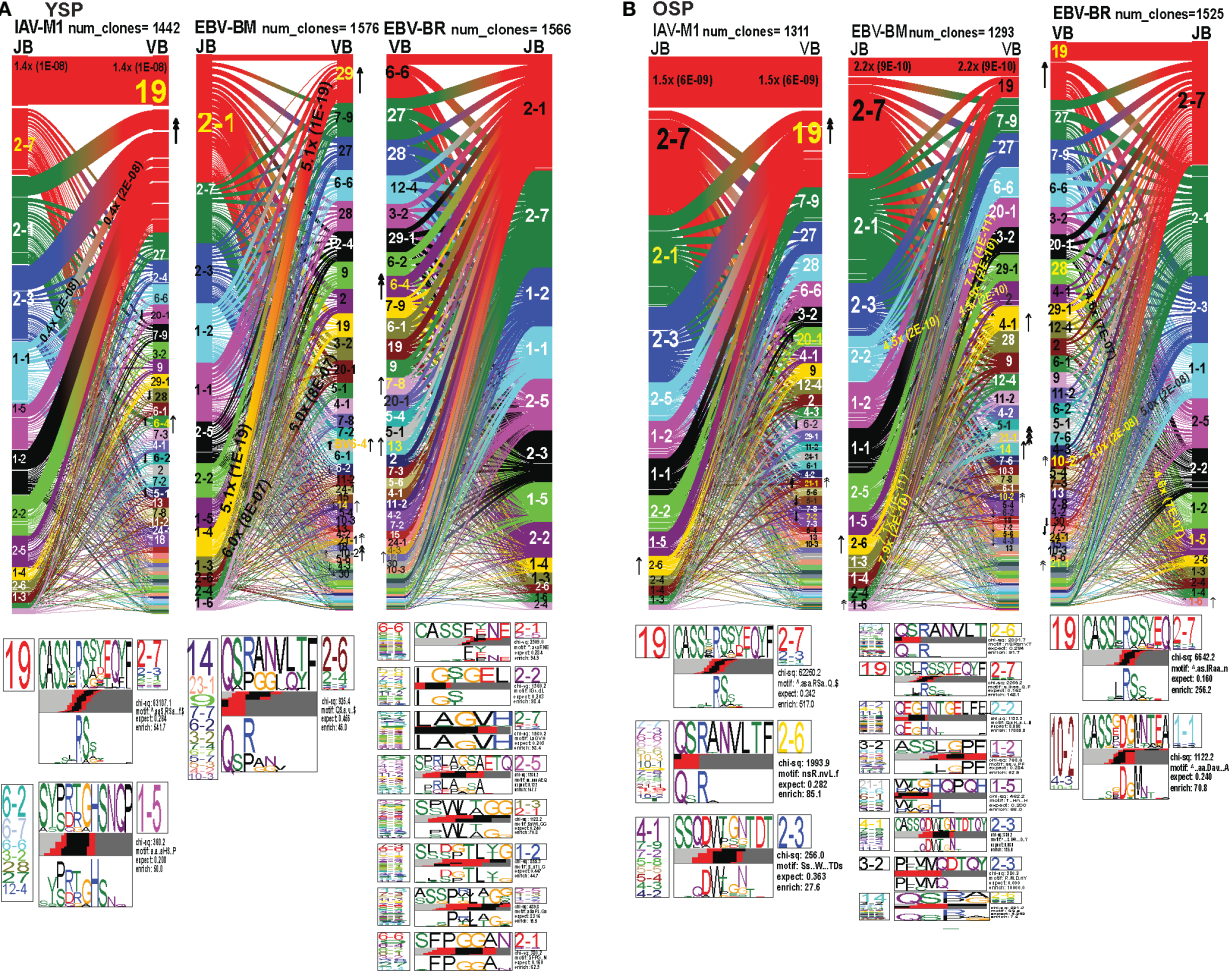
TRBV and TRBJ gene pairing and CDR3 motifs for IAV-M1, EBV-BM and EBV-BR CD8 T cell populations differ between young and older. This was determined after TCR high throughput sequencing of tetramer-sorted CD8 T cells in Y and O donors (Y, n=4; O, n=4-5) using ribbon-plot analyses. Ribbon plots show patterns of TCR V-J pairings in TRBV in young **(A, B)** older donors (num_clones, indicates the number of clones analyzed). Genes are colored by frequency within the repertoire with red>green>blue>cyan>magenta>black. The arrows indicate fold increase usage of those V or J regions compared to naïve random repertoire suggesting antigen-driven expansion (no. of arrow heads are log_2_). Underneath each ribbon plot are the unique clearly defined CDR3 motifs of TRBV repertoire of the indicated antigen-specificity. There can be multiple different CDR3 motifs for any one specificity. For each CDR3 motif, the upper motif 1 (labeled Mf1 in young IAV-M1 as a representative) shows the amino acids which are enriched in comparison to the total tetramer+ population of that specificity; the lower motif 2 (labeled Mf2 in young IAV-M1 as a representative) shows the amino acids which are enriched compared to a naïve random CD8 T cell repertoire. Both indicate that the identified amino acids are important for an antigen peptide/MHC contact. Naive repertoires do not generate motifs as this requires the presence of clonal expansions. (analysis method from Dash et al. (6); Kamga et al. (8). The text within the bars joining particular *BV* and *BJ* gene regions indicate the fold increased usage of that pairing (and statistical significance) compared to a naïve random TCR repertoire. Bar in between the Mf1 and Mf2 depicts which part of the CDR3 is derived from the V (light grey), N (red), D (black) and J (dark gray) regions.

**Figure 8:**
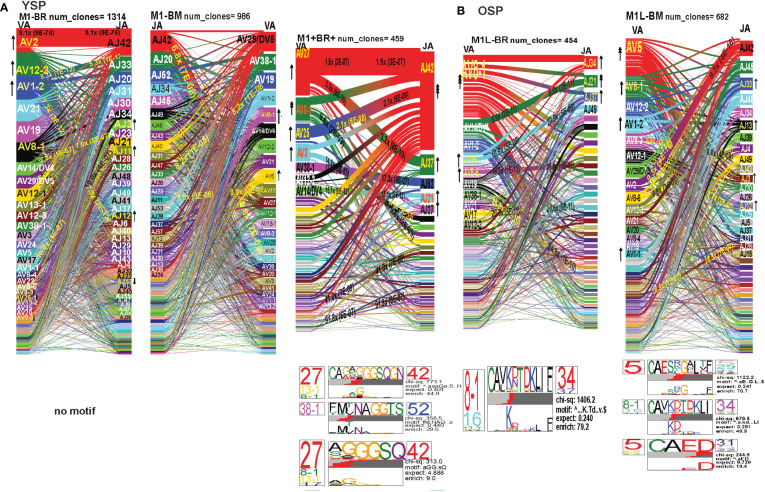
The hierarchy of AV and AJ gene pairing and CDR3 motifs of cross-reactive M1BM, M1BR, and M1+BR+ CD8 T cell populations differ from cognate and between young **(A)** and older donors **(B)**. This was determined after TCR high throughput sequencing of tetramer-sorted CD8 T cells in Y and O donors (Y, n=4; O, n=4-5) using ribbon-plot analyses (6, 8). The figure legends of **Figures 6**, **7**, provide a detailed description of the ribbon-plot analyses that is applicable to this figure. M1BM or M1BR are cross-reactive EBV-BM or EBV-BR tetramer+ cells sorted from IAV-M1 stimulated short term cultures. M1+BR+ are double tetramer+ co-staining CD8 T cells sorted from the IAV-M1 short-term culture.

**Figure 9:**
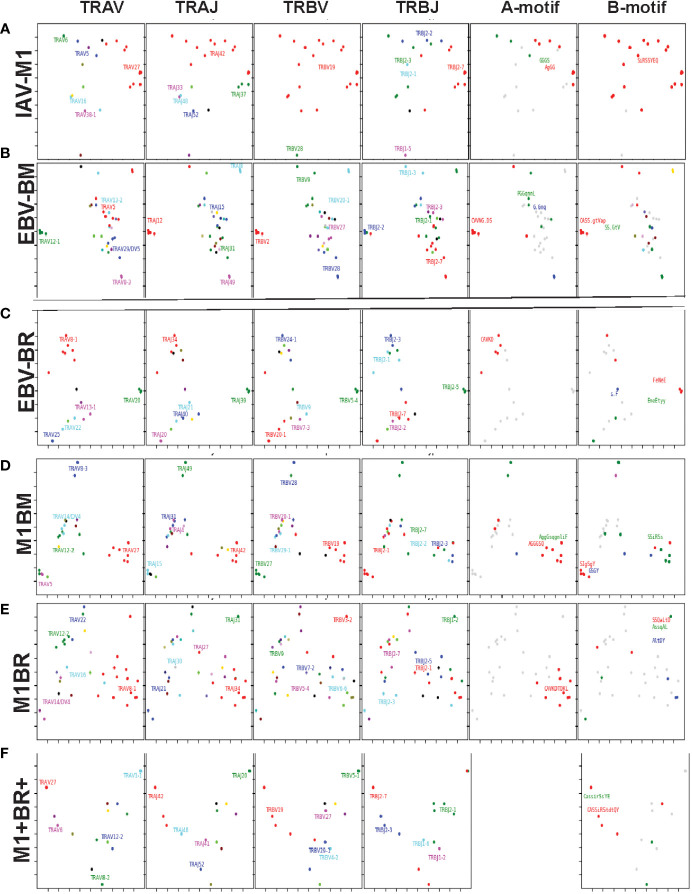
The hierarchy of BV and BJ gene pairing and CDR3 motifs of cross-reactive M1BM, M1BR, and M1+BR+ CD8 T cell populations differ from cognate and between young **(A)** and older donors **(B)**. This was determined after TCR high throughput sequencing of tetramer-sorted CD8 T cells in Y and O donors (Y, n=4; O, n=4-5) using ribbon-plot analyses (6, 8). The figure legends of **Figures 6**, **7**, provide a detailed description of the ribbon-plot analyses that is applicable to this figure. M1BM or M1BR are cross-reactive EBV-BM or EBV-BR tetramer+ cells sorted from IAV-M1 stimulated short term cultures. M1+BR+ are double tetramer+ co-staining CD8 T cells sorted from the IAV-M1 short-term culture.

**Figure 10:**
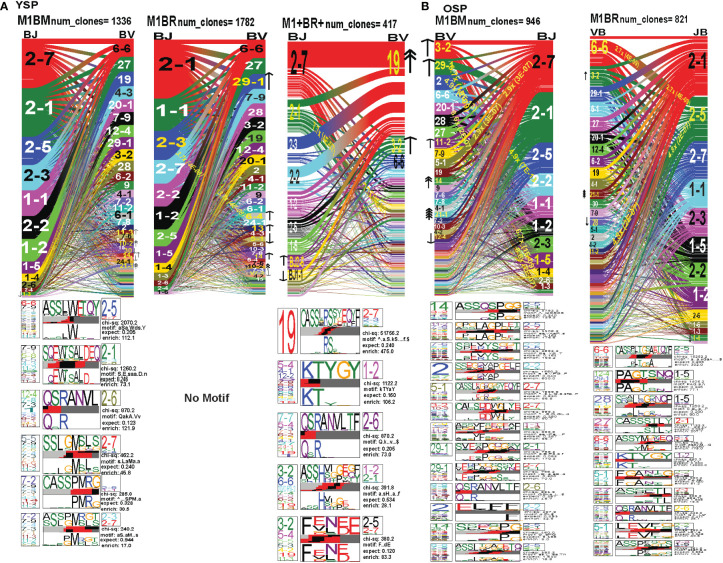
Kernel Principle Components Analysis of single cell TCRab sequencing shows that the cross-reactive populations differ from cognate, at times using combinations of TCR features specific for the two different ligands **(A–F)**. Tetramer-sorted single cell CD8 T cells from representative Y and O donors were transcribed into cDNA, then amplified *AV/AJ* and *BV/BJ* gene combinations using primers from a published multiplex PCR technique (6, 8). TCR single cell sequencing data was combined from Y and O donors (Y, n=2; O, n=2). Kernel Principal Components Analysis (kPCA) 2D projection plots were used to show the AV/AJ/BV/BJ pairing of the single analyses (6). Each point on the plot represents a single TCR clone, the location of the clone is based on TCRdist measurements which place similar TCR clones closer together on the 2D plot. Each clone can be tracked to determine the gene usage and pairing by the color and location. Each of the four gene segments, TRAV, TRAJ, TRBV, and TRBJ (left to right) has a separate plot. The last two plots, represent the CDR3 motif generated for TRAV/AJ and TRBV/BJ genes. (for details on sequences see **Tables 1** and **S4**).

To examine changes in TRAV usage between older and young donors in more detail we performed TCRdist quantitative analyses using the top 400 clonotypes by frequency in IAV-M1, EBV-BM and EBV-BR-specific TCR repertoires. TCRdist analysis quantifies clusters of TCRs with similar features, enabling the visualization and dimensionality of these clusters on a 2D projection of the TCRdist landscape (6). The distance between 2 or more TCRs is calculated using a similarity-weighted Hamming distance, based on amino acids in the CDR loops that contact pMHC. A gap penalty is based on variations in CDR length and the CDR3 loop is given a higher weight as it is primarily responsible for antigen-specific recognition (6). In the original TCRdist analysis program, epitope-specific single cell TCR sequencing data can be presented as ribbon plots which show patterns of TCR AV/AJ/BV/BJ pairings (num_clones, indicates the number of clones analyzed). Genes are colored by frequency within the repertoire with red>green>blue>cyan>magenta>black (6). The arrows indicate fold increase usage of those V or J regions compared to naïve random repertoire suggesting antigen-driven expansion (no. of arrow heads are log_2_) (6). The CDR3 motif analysis in this program, enables the determination of which amino acids are commonly used in certain positions of the CDR3, indicating that they may be important for antigen recognition based on the enrichment of certain amino acids when compared to a naïve background. The CDR3 motif analysis generates two motifs, motif 1 shows the amino acids which are enriched in comparison to the total tetramer+ population of that specificity; motif 2 shows the amino acids which are enriched compared to a naïve random CD8 T cell repertoire (6). Here, we have adapted the TCRdist program to analyze high throughput TRAV or TRBV sequences (**Figures 6**–**10**; **Supplemental Table S2**).

As seen in **Figure 6**; **Supplemental Figure S2**, **S4** and summarized **Table S2** there were differences in the pattern and specific TRAV and TRAJ gene usage in IAV-M1, EBV-BM and EBV-BR responses of O and Y donors consistent with the family usage data (**Figure 4**).

In the IAV-M1 repertoire, the *AV/AJ* gene pairing analyses showed older like the young, retained enriched usage of certain significant *AV/AJ* gene pairings such as the public TRAV27/J42 (2x greater than naïve repertoire), AV38/AJ52 (8x), plus the less commonly described TRAV25/AJ42 (3x) (**Figures 6A, B**). However, the young had some atypical *AV/AJ* gene pairings not observed in older donors including V2/AJ42 (3.4x), AV27/AJ37(4.8x), AV1.2/AJ33(7.8x) and AV1.2/AJ12(13x). The older had enhanced usage of TRAV12.2, 8.6, and 24 which was not observed in young. Both older and young used the public AV27-GGGSQ-JA42 CDR3a motif, but the older did not maintain the public CDR3a motif AV38-FMxNAGGT-J52, that was observed in young. Instead, older retained TCR with atypical AV families paired with AJ42 containing variations of the public motif like, AV12.1/AV12.2/AV8.1- NxGGGSQ-TRAJ42 and AV12.2/8.1/2/5-NGGGSQ-AJ42. Interestingly, in all 3 epitope-specific responses, AJ42 gene usage was increased above random naïve repertoire. These data suggest AJ usage could enhance or contribute to the cross-reactivity that exists between these 3 epitopes.

In EBV-BM repertoire, older donors used only 2 dominant AV retaining the public AV5 and AV12.2 family and 3 dominant AJ, including AJ24,11, and 12, while young used 4 dominant AV including AV5, 12.2, but also AV2, 1.2 and 2 dominant AJ in common with older, including AJ11, 12 but also used AJ42, 20, 30 (**Figures 6A, B**). The *AV/AJ* gene pairing analyses showed older donors, retained enriched usage of the public AV5/AJ31 (5.4x), as well as unique AV14/DV4/J24 (2x) and AV2/AJ42(6.6x) (also present in IAV-M1), and was the only one in common with young donors who used it at 8x above the naïve random TCR repertoire. The young had some atypical *AV/AJ* gene pairings not observed in older including, AV5/AJ37(5x), AV1.2/A31(6.5x), AV1.2/AJ12(5.5x), AV12.1/AJ12(6.88x), and AV12.3/AJ52(19x). Both young and older used the public CDR3a motif, AV5-CA(E/D)DxNARLM-AJ31. The older also used a new CDR3a motif AV14-CAMRGGGMT-J42.

In EBV-BR repertoire older and young donors retained increased usage of the public AV8.1 and AV12.2 paired with multiple different AJ families (**Figures 6A, B**), further supporting our earlier observations that TRAV8.1 plays a major role in EBV-BR TCR repertoire selection (7). However, older had lost the public AV8.1/8.3/16/12.2-VKDTDKL-J34 and in fact had no identifiable CDR3a motif. It should be noted that this motif in young can associate with multiple different AV besides AV8.1. This lack of a public motif is highly suggestive of more variable repertoires or private repertoires between older donors. The AV/AJ gene pairing analyses showed older and young had enriched usage of AV2/AJ42 (7-8x), which was also used by the IAV-M1 and EBV-BM responses of both groups. The older also had increased usage of TRAV27/AJ42(5x), which is usually associated with being a public repertoire feature in IAV-M1 responses. The AJ gene usage was unique for older and young, but they both did have a dominant AJ42(2x) usage, as they did in IAV-M1 and EBV-BM responses. As mentioned earlier, AJ42 is one of the public features used by IAV-M1 responses (43).

Overall, these data suggest that while the classical public TRAV and TRAJ genes were being used for all 3 epitope-specific repertoires, there are significant differences in both AV and AJ usage and pairing in young and older donors. The overlap in certain gene usages between epitopes would increase the potential for TCR cross-reactivity.

### *TRBV* and *TRBJ* gene usage, pairing and CDR3 motifs of IAV-M1, EBV-BM and EBV-BR differ between young and older donors

As seen in **Figures 7A, B**; **Supplemental Figures S3**, **S5** and summarized **Supplemental Table S2** there were differences in the pattern and specific *TRBV* and *TRBJ* gene usage in IAV-M1, EBV-BM and EBV-BR responses of older and young donors. Perhaps not surprisingly, in the IAV-M1 TRBV repertoire, both older and young donors maintained a significantly greater usage of the public BV19(4x) the public BV19/BJ2.7(1.5x) in comparison to the naïve random TCR repertoire (**Figures 7A, B**). However, the older had increased usage of the atypical BV21.1(4x), while young increased usage of the atypical BV6.4(2x). Older donors also showed a significant enrichment of BJ2.6(2x) usage. Both older and young donors used the public CDR3b motif BV19-CASSIRSSYEGY-J2.7/2.3/2.1. Interestingly, this same motif but restricted to BV19/J2.7 pairing begins to dominate in the EBV-BM and EBV-BR TCR repertoires of the older but not the young. There appears to be enriched usage of ‘IRSS” in the EBV-BM and BR repertoires of the older as compared to ‘xRSx” in the IAV-M1 response. Older donors had another dominant CDR3b motif, V7.7/7.3/6.6/5.6/10.3/10.2/21.1-QSRANVLTF-J2.6, accounting for the enrichment of the unusual BJ2.6 usage in the older donors. This same motif although not present in IAV-M1 repertoires in young was the most dominant motif in the EBV-BM TRBV repertoire of the young (although associated with different BV) and the older. This is highly suggestive that this particular TRBV motif may be selected into the IAV-M1 TCR repertoire because of cross-reactivity with EBV-BM. There was a second novel dominant CDR3b motif in the older BV4.1/17/9/7.2/5.8/5.4/4.3/4.2-SSQDWTGNTDT-J2.3, which was largely selected on BJ2.3 paired with multiple different BV. This same motif was also present in the EBV-BM repertoire of older but not young donors.

In the EBV-BM TRBV repertoire, there has been a complete shift in BV/BJ dominance hierarchy in older as compared to young donors(**Figures 7A, B**). The older showed an increased usage of BV19/BJ2.7(2.2x) pairing (public for IAV-M1 responses), which is a non-canonical pairing for EBV-BM responses in young, middle-aged donors or in AIM (7, 9). In comparison, young preferentially used the public gene pairings, BV29.1/BJ1.4(5x) and BV20.1/BJ1.3(5x). The older retained usage of the public BV20.1/BJ1.3(7x). However, they also had increased usage of BV2/BJ2.2(4.5x), and BV3.2/1.4(7.9x) pairings, with increased usage of BV14(2x), and less typical BV4.1(2x), BV10.2(4x) and BV21.1(8x). Young donors also had increased usage of BV 21.1(2x), 10.2(2x), as well as, BV6.4(2x) (not increased in older). For EBV-BM TRBV repertoire, older had 8 unique CDR3b motifs never previously described, that were generated using several different BV, while young donors had one predominant CDR3b motif. The CDR3 motif “QRANLVLT,” which was generated with BJ2.6 associated with multiple BV was the dominant motif for young, but the public motif, “QSPGG” associated with BV14 was also present co-mingled within the other motif. As noted above the older CDR3b motifs contained strong overlaps with IAV-M1 motifs, including multiple(x)BV-QRANLVLT-JB2.6 BV19-CASSIRSSYEQY-27, multiple(x)BV-SSQDWTGNTDT-BJ2.3 suggesting these may be selected to dominate because of TCR cross-reactivity.

The EBV-BR TRBV repertoire had also completely shifted in TRBV/BJ dominance hierarchy in older as compared to young (**Figures 7A, B**). Like EBV-BM, older had a dominant usage of the TRBV19/BJ2.7 pairing. In contrast, young used multiple different BV relatively equally but TRBV6.4, 7.8, 13 and TRBV14 were significantly above the naïve repertoire. In contrast, older donors showed an increase usage of BV19, 10.2, and 21.1. Only older showed an increased usage of the BJ1.6 gene. Older donors also had an increased usage of BV28/BJ1.5(4.6x) and BV10.2/BJ1.1(5x). Young donors used 8 different CDR3b motifs, where the BJ portion appeared to be important in selection, while older had two major CDR3b motifs. The CDR3b motifs do not have a dominant BV, but instead the BJ dominated including BJ2.1 and BJ2.7 usage. In the older the most dominant motif was the IAV-M1 public BV19-CASSIRSSYEQY-27. The second older motif was a unique, V10.2/4.3/10.1-CASSxDGMNTEA-J1.1.

Overall, these results strongly suggest that as the TCR repertoire narrows in older they are retaining TCR that are cross-reactive between two very common human viruses IAV and EBV, that we are exposed to frequently, one with recurrent infections and the other a persistent virus, which frequently reactivates.

### The hierarchy of *TRAV* and *TRAJ* gene usage, pairing and CDR3 motifs of cross-reactive M1BR, M1BM and M1+BR+ CD8 T cell populations are unique and differ between young and older donors

In order to examine whether and how cross-reactivity might influence or change TCR repertoire with increasing age we assessed both types of cross-reactive CD8 T cells, single tetramer+ and double tetramer+ from IAV-M1 peptide stimulated short term cultures (23, 26). In order to examine single tetramer+ cross-reactive CD8 T cells we sorted EBV-BM (M1BM) or EBV-BR (M1BR) tetramer+ cells from IAV-M1 stimulated short term cultures for TCR high throughput sequencing. We also sorted M1+BR+ double tetramer+ cells from the IAV-M1 stimulated short-term cultures of two young donors who had this population. As seen in **Figures 8A, B** and summarized Supplemental **Supplemental Table S2** there were differences in the pattern and specific TRAV and TRAJ gene usage in the cross-reactive vs their cognate counterpart in each donor group suggesting they are unique populations with their own characteristics that make them capable of responding to two different antigens. However, like the cognate repertoires the M1BR and M1BM repertoires of older vs young donors differ suggesting that the older are retaining or developing a particular subset of cross-reactive T cells.

The M1BR TRAV repertoire in older dramatically differed from the cognate EBV-BR. Older had increased usage of AV8.1(8x) (public AV for EBV-BR) and this could pair with many different AJ that also showed increased usage including AJ34(2x), AJ21(4x) or AJ37(2x). This contrasted with the EBV-BR repertoire, VA 12.2(2x) and AV8.1(2x) usage were co-dominant and they paired with so many different AJ that none was dominant. In older all of the other features of TCRAV usage were unique to the cross-reactive M1BR as compared to the cognate. These included increased usage of AV8.6-AJx(18.9x), AV16-AJ49(10.9x), AV14-DV4-AJ21(6.9x), AV38.1-AJx(24.8x) (associated with IAV-M1), and V17-AJx(20.6x). Curiously, the cross-reactive M1BR, unlike the cognate EBV-BR, had a dominant CDR3a motif, AV8.1-CAxKxTDKLIF-AJ34/37, which although not identical is reminiscent of the public EBV-BR motif seen in young and described in AIM donors (7). This might suggest that IAV-M1 cross-reactivity even in AIM leads to the selection of these dominant clonotypes. These results suggest that there are unique AV/AJ pairings that lead to cross-reactive responses that may be more stringent than cognate EBV-BR.

In contrast, the young cross-reactive M1BR response maintains many of the same AV usage as the young cognate EBV-BR including AV2(2x), AV12.2(2x), AV1.2(2x). Young had increased usage of the atypical AV2-AJ42(9x) for M1BR, which appeared previously in all 3 epitope-specific (cognate) responses of the young, but is not present in the older. Young donors showed unique pairings as compared to EBV-BR, such as, AV17-AJ21(10x), AV1.2-AJ33(7.3x), AV1.2-AJ12(7.3x), AV14-DV4-AJ21(6.9x) (also present in older). Curiously, in contrast to the older the young M1BR population did not yield a CDR3 motif while their cognate EBV-BR had the public AV8.1-VKDTDKL-J34. These results might suggest that the older overtime have selected more skewed, and narrow cross-reactive M1BR responses with public features compared to the young donors.

However, in the young donors we also had the unique cross-reactive double tetramer M1+BR+ population that was only isolated in two of four young donors. In the M1+BR+ repertoire there was increased usage of AV27 (2x)(like IAV-M1), AV8.1(4x)(like EBV-BR), AV25(2x)(unique) and AV3(2x)(unique), as well as AJ42(8x)(like IAV-M1), AJ37(2x)(unique), AJ21(2x)(unique) and AJ27(2x)(unique). The most dominant pairings were AV27/AJ42(1.5x) (public for IAV-M1), AV8.1/AJ37(9.6x) (public for EBV-BR), AV27/AJ37(3.2x)(unique), AV25/AJ42(2x)(unique), AV3/AJx(15x)(unique), AV38/AJ52(17.3x)(public for IAV-M1), and AV14/DV4/AJ21(15x)(unique). Two of the CDR3 motifs, exhibited glycine runs, “xGGGx,” (AV27/13.1/8.1CAGx(G/S)GGGSQGNJ42) and (AV27/8.1/13.1/17-(A/S)GGGSQ/J42) and (AV38.1-FMxTNAGGTS/52) and were variants of public motifs in IAV-M1 repertoires. This double tetramer+ population appears to combine features of both cognate responses, as well as having unique features.

In the M1BM repertoire the AV and AJ usage differed from cognate EBV-BM and IAV-M1 in the older, except in the increased usage of AV5(4x) (public for EBV-BM) paired with many different AJ, AV8.1(2x) (public for EBV-BR) and AJ12.2(2x)(used by EBV-BM and EBV-BR). There was increased usage of unique pairings AV8.3-AJ49(9.3x), AV8.6-AJ4(9.8x) and AV12.1/AJ12(8.8x). One of the most dominant pairings once again was AV2-AJ42(9.3x) (also seen in young). The 3 CDR3 motifs that were generated contained AV5-AJ31 and AV8-AJ34 pairings and differed from those observed in the cognate EBV-BM response. The AV5-CAED-AJ31 motif which was identified is perhaps a variant of the public EBV-BM motif AV5-xEDNNAx-AJ3. A second motif was unique AV5-CAESxGxLxF-AJ35/29/37. The AV8.1/16/1.1-CAVKDTDKLI-AJ34/J23 motif is a variant of the public EBV-BR motif.

Overall, these data suggest that young donors had most likely such private diverse cross-reactive TCR repertoires that no motifs were identified for either M1BR or M1BM. In contrast, it would appear that older donors are most likely retaining selected cross-reactive TCR that have been stimulated by both antigens at some point leading to clonal expansions and identifiable public features. The results also suggest rather logically, that a TCR that has some features of either cognate response may be more likely to be cross-reactive. However, these cross-reactive responses can also have totally unique public features, while displaying minor features if any of the cognate responses. We will use single cell sequencing to determine whether the TCR AV/AJ/BV/BJ gene pairings in cross-reactive responses will demonstrate a combination of public repertoire features of IAV and EBV (i.e. M1BR, AV8.1/AJ34 and BV19/BJ2.7). The single cell data will allow the determination of factors/features that may provide a mechanism by which TCR cross-reactivity can occur.

### The hierarchy of TRBV and TRBJ gene usage, pairing and CDR3 motifs of cross-reactive M1BR, M1BM and M1+BR+ CD8 T cell populations are unique and differ between young and older donors

In the M1BR TRBV repertoire, older had a significant increase in unique BV/BJ pairings as compared to IAV-M1- or EBV-BR-specific responses, such as, BV6.6-BJ2.5(2.7x), which had a unique CDR3 motif BV6.6(x24BV)-CASSPLTGAETQF-BJ2.5/2.3/1.1, BV11.2-BJ2.5(4.8x), BV3.2(2x) which had a unique CDR3 motif BV3.2(x9BV)-KTYGY-J1.2. Also, there was an increased selection of the atypical BV21.1 with an 8-16-fold increase in all responses of the older including all 3 cognate epitope responses and the M1BM and M1BR cross-reactive responses. Unlike the cognate EBV-BR in either older or young there were 11 different distinct CDR3b motifs that were predominantly unique in older. This would suggest that there are more stringent requirements for cross-reactive M1BR TCRb than cognate EBV-BR, which is largely selected on TRAV. These CDR3 motifs were largely derived from the N region. They appeared to have highly variable TRBV usage, which was associated with particular TRBJ suggesting that the BJ region may play a significant role in specificity and selection of these cross-reactive TCR. They did have the CDR3b motif BVx-QSRANVLTF-BJ2.6, which was common to IAV-M1, EBV-BM, and M1BM repertoires in older and dominant in young EBV-BM. Older in M1BR and young in M1+BR+ responses had the CDR3 motif BVx-KTYGY-BJ1-2 which was not seen in cognate responses.

In young donors, the M1BR repertoire had increased usage of BV29.1(2x)(public for EBV-BM). There also was a significant 3.7-fold increase in the unique BV6.4/BJ2.3. Once again indicating the importance of TRBV in selection of EBV-BR cross-reactive TCR, the young donors had increased usage of several BV including, BV13(2x), BV14(2x), and BV10.2(4x). Young donors did not have a CDR3 motif. The lack of CDR3 motifs as compared to older donors might as with TRAV/AJ relate to the higher diversity and private nature of cross-reactive responses in young.

The M1+BR+ repertoire in the two young donors, had increased usage of BV19(4x), (public for IAV-M1). As seen in the pairing for IAV-M1, in M1+BR+, BV19 is most commonly paired with BJ2.7. There were increases in unique BV3.2(2x), BVx-BJ2.1(7.7x) and BJ2.6(2x) usage. The most dominant CDR3 motif was BV19-CASSIRS(S/T)YEQYF/-2.7/2.3/2.2, which is most commonly used in IAV-M1 responses consistent with this TCRBV motif playing a role in TCR cross-reactivity (see also Single cell sequence **Table 1**). There was also another unique CDR3 motif BV3.2/5.4/5.5/4.2/19/11.3-F9E/V)N(E/D)E-J2.5/2.7 most likely specific for cross-reactive responses (see Single cell sequence **Table 1**).

**Table 1:** Unique TCR public features (VJ usage and CDR3 motifs) of cross-reactive vs cognate single cell clones.

Epitope specificity	CLONE ID	AV	CDR3a	AJ	BV	CDR3b	JB	No.
**A. IAV-M1**	ES179M1-04	27*01	CAAGGSQGNLIF	42*01	19*01	CASSIRSSYEQYF	2-7*01	1
	ES556M1-01	5*01	CAETGGGSQGNLIF	42*01	19*01	CASSIRSSYEQYF	2-7*01	1
	ES556M1-02	27*01	CAGGGSSNTGKLIF	37*02	19*01	CASSIRSSYEQYF	2-7*01	1
	ES556M1-14	27*01	CAGASGNTGKLIF	37*01	19*01	CASSIRSSYEQYF	2-7*01	1
	ES556M1-16	27*01	CAGGGSQGNLIF	42*01	19*01	CASSIRSSYEQYF	2-7*01	1
	ES556M1-17	27*01	CAGGGSSNTGKLIF	37*02	19*01	CASSIRSSYEQYF	2-7*01	1
	D044M1-04	27*01	CAGGGSQGNLIF	42*01	19*02	CASSIRSSYEQFF	2-7*01	4
	D044M1-18	27*01	CAGGGSQGNLIF	42*01	19*02	CASSIRSSYEQYF	2-7*01	11
	D044M1-22	13-1*02	CAPSGGGSQGNLIF	42*01	19*02	CASSIRSSYNEQFF	2-7*01	3
	ES556M1-07	27*01	CAGVDGGSQGNLIF	42*01	19*01	CASSIRSSYEQYF	2-7*01	1
	ES556M1-08	16*01	CARKSYGQNFVF	26*01	19*01	CASSIRSSYEQYF	2-7*01	1
								
** M1BM**	ES556M1BM-03	27*01	CAGGGSQGNLIF	42*01	19*01	CASSIRSSYEQYF	2-7*01	1
	ES556M1BM-09	27*01	CAGGGSQGNLIF	42*01	19*01	CASSIRSSYEQYF	2-7*01	1
	D044M1BM-08	27*01	CAGGGSQGNLIF	42*01	19*01	CASSIRSSYEQFF	2-7*01	1
** BRM1**	ES179BRM1-05	27*01	CAGGGSQGNLIF	42*01	19*02	CASSIRSSYEQYF	2-7*01	9
	ES179BRM1-09	27*01	CAGGGSQGNLIF	42*01	19*02	CASSIRSSYEQYF	2-7*01	1
** M1+BR+**	ES179M1+BR+10	27*01	CAGGGSQGNLIF	42*01	19*02	CASSIRSSYEQYF	2-7*01	1
	ES179M1+BR+12	27*01	CAGGGSQGNLIF	42*01	19*02	CASSIRSSYEQYF	2-7*01	2
	D044M1+BR+02	27*01	CAGGGSQGNLIF	42*01	19*02	CASSIRSSYEQYF	2-7*01	2
								
** B. IAV-M1**	ES556M1-04	38-2/DV8*01	CAYSSSAGGTSYGKLTF	52*01	19*01	CASSIGLYGYTF	1-2*01	1
	D044M1-10	38-2/DV8*01	CAYMINAGGTSYGKLTF	52*01	19*02	CASSIGVYGYTF	1-2*01	1
	D044M1-01	38-2/DV8*01	CAYSPNAGGTSYGKLTF	52*01	19*02	CASSMGLYGYTF	1-2*01	2
								
** EBV-BM**	D044BM-09	5*01	CAEPRDSNYQLIW	33*01	27*01	CASIGSGYPYNEQFF	2-1*01	13
	D044BM-12	29/DV5*01	CVYRNSNARLMW	31*01	27*01	CASIGSGYPYNEQFF	2-1*01	1
								
** M1BM**	D044M1BM-07	12-2*01	CAVNNQAGTALIF	15*01	27*01	CASIGSGYPYNEQFF	2-1*01	1
	D044M1BM-14	12-2*01	CAVNSQAGNALIF	15*01	27*01	CASIGSGYPYNEQFF	2-1*01	1
	D044M1BM-20	38-2/DV8*01	CAYSPNAGGTSYGKLTF	52*01	27*01	CASIGSGYPYNEQFF	2-1*01	1
	D044M1BM-05	5*01	CAEPRDSNYQLIW	33*01	27*01	CASIGSGYPYNEQFF	2-1*01	1
	D044M1BM-22	5*01	CAEPRDSNYQLIW	33*01	27*01	CASIGSGYPYNEQFF	2-1*01	5
	D044M1BM-19	5*01	CAEPRDSKYQLIW	33*01	27*01	CASIGSGYPYNEQFF	2-1*01	1
** BRM1**	D044BRM1-05	5*01	CAEPRDSNYQLIW	33*01	27*01	CASIGSGYPYNEQFF	2-1*01	5
	ES179BRM1-08	38-1*01	CAFMTNAGGTSYGKLTF	52*01	19*02	CASSQGSHGYTF	1-2*01	1
** M1+BR+**	D044M1+BR+03	24*01	CAPNSGYSTLTF	11*01	27*01	CASIGSGYPYNEQFF	2-1*01	1
								
**C. M1BM**	ES556M1BM-08	27*01	CAGGGSQGNLIF	42*01	14*01	CASSQSPGGTGTF	2-7*01	1

Red text highlights motifs. Color blocking highlights similarities.

The M1BM TRBV repertoires of both older and young are very different in hierarchy and usage from each other and from the cognate EBV-BM. In older there was increased usage of BV3.2(2x), BV11.2(2x) and BV29(2x)(public for EBV-BM), 29.1/BJ1.4(6.7x), BV2/J2.2(4.9x), BV20.1/J1.3(8.6x) and BV5.1/2.7(2.9x). The most widely used BV amongst most epitope responses for older, BV21.1, was increased 16-fold. The most common CDR3 motif was BV14(x9BV)-ASSQSPGG-J2.5/2.1/1.1/2.2/2.6/2.4, which is a variant of the public EBV-BM motif. The CDR3 motif “QSRANVTL” was associated with BJ2.6 usage and was present in the cognate EBV-BM responses as well M1BM responses for older. The BJ usage appeared to be the most dominant specificity and selection factor of the CDR3 motifs. In contrast, young had increase usage of BV14(2x) (public for EBV-BM). There was increased usage of unique BV including BV11.3(2x) and BV10.2(4x). BV21.1 found in several other responses in older usage was increased 16-fold. There were 4 unique CDR3 motifs which largely differed from older and young cognate EBV-BM and Y M1-BM except for the BVx-QSRANVLTF-BJ2.6. There appeared, as with M1BR, to be stringent requirements in BV and BJ usage as well as CDR3 motifs in the cross-reactive M1BM TCR in both young and older donors. This only makes sense as the cross-reactive TCR has to recognize two different epitopes, while cognate-specific TCR are only have to recognize one epitope.

### Single cell TCR sequencing and TCR cross-reactivity

Since we postulate that TCR cross-reactivity is playing a role in repertoire evolution with increasing age we more closely examined TCR repertoire of cognate and cross-reactive tetramer-sorted CD8 T cells at the single cell level. For these studies as we were addressing features of cognate and cross-reactive TCR we pooled the data of young and older donors. We were interested in addressing two particular questions. First, we wanted to determine if there was evidence that the TRBV ‘IRSS” motif expressing clones, which are public for IAV-M1 repertoires were actually preferentially selected by the cross-reactivity with the EBV epitopes with increasing age as is suggested by our high throughput sequencing data. Second, we wanted to determine if there was any evidence that the cross-reactive clones had TCR features that would increase their ability to interact with two antigens. For this type of comparison the Kernel Principal Components Analysis (kPCA) 2D projection plots which show the AV/AJ/BV/BJ pairing of the single cell analyses (6) was highly useful. Each point on the plot represents a single TCR clone, and the location of the clones is based on TCR dist measurements placing similar TCR clones closer together on the 2D plot. Each clone can be tracked to determine the gene usage and pairing by the color and location.

At a glance it is clear that the characteristics including CDR3 motifs and distributions of the TCR clones are unique for each epitope and for each of the cross-reactive populations (**Figure 9**, **Supplemental Table S4**). The cognate IAV-M1, EBV-BM and EBV-BR had many characteristics that have been previously identified and shown in the high throughput sequencing data. It should be noted that these single cell studies identified a new EBV-BM specific TRAV motif AV-12.1-CVVNGxDS-AJ12.1. It appeared to pair with TRBV motif TCRBV2-CASS.GtVap-BJ2.2. The pKCA analyses (**Figure 9**) and **Table 1** showing the single cell sequences of cross-reactive clones that are M1BR, M1+BR+ and M1BM, as well as BRM1 specific, and contrast them to clones with some similar features, if there are any in IAV-M1, EBV-BM, EBV-BR-specific, demonstrate findings compatible with our hypothesis that certain TCR clones are preferentially retained in the older due to TCR cross-reactivity. For instance, there is a clear selection for clones specifically expressing AV27-CAGGGSQGNLIF-AJ42 paired with BV19-CASSIRSSYEQY-JB2.7/2.1 in the M1+BR+, BRM1 and M1BM cross-reactive populations suggesting that this unique clone which dominates the EBV-BM and EBV-BR TCR repertoires of older donors has some ability to interact with all 3 epitopes (**Table 1A**). It may be at differing affinities to the different epitopes which might make it difficult to derive a crystal structure to determine exactly how it interacts with EBV-BM and EBV-BR, although it does appear to bind EBV-BM and EBV-BR tetramers. If this type of clone which is most likely not optimum for EBV control begins to dominate the EBV-BM and EBV-BR TCR repertoires in older donors they may have difficulty controlling this persistent virus, perhaps enhancing chances of developing EBV-associated cancers (27).

There is also a new unique cross-reactive TCR that predominates in the M1BM repertoire AV5-CAEPRDSNYQLIW-J33.1 paired with BV27-CASIGSGYPYNEQFF-2.1, where the AV5 usage is public for EBV-BM and the motif could be a variant that could recognize EBV-BM, while the BV27 has been shown in our studies to be used by all three epitope-specific responses, but this clone contains a CDR3b motif reminiscent of the public IAV-M1 BV19-xGxY-J2.1 (3) as shown in the **Table 1B**, which obligately pairs with AV38/AJ52 in the cognate IAV-M1 response. A second cross-reactive M1BM clone has a public IAV-M1-specific TCRa, AV27-CAGGGSQGNLIF-42 paired with a public EBV-BM-specific TCRb, BV14-CASSQSPGGTGTF-2.7. This clone is EBV-BM tetramer+ but able to proliferate in response to IAV-M1 peptide. Without an appropriate TCRb, it is most likely low affinity to IAV-M1 and unlikely to bind IAV-M1 tetramer, but could easily proliferate during an acute IAV infection like it did in the IAV-M1 stimulated short term culture, yet not being an optimum response to protect against IAV infection.

The cross-reactive M1BR repertoire, had increased usage of clones expressing the public EBV-BR-specific AV8.1-CAVKDTDLIF-AJ34 (or variants of it) paired with multiple different TCRb chains some of which did express the IAV-M1 public BV19 family (**Table 1D**). Once again, as these M1BR clones are high affinity to EBV-BR, but low affinity to IAV-M1 it would not be ideal for them to start to proliferating during acute IAV infection. There was one M1+BR+ clone which did stain with both tetramers, AV16-CALKDTDKLIF-AJ34 paired with BV25-CASSEWFSYNEQFF-BJ2.1 which might be interesting for future crystal structure studies to determine exactly how this TCR can interact with both ligands (**Table 1D**). There are also other completely unique clones without public features that are able to bind both tetramers that could be used for crystal structure studies (**Table S6**). There was also a relatively unique public M1BR cross-reactive TCR which expressed the public EBV-BR AV8.1 with a relatively unknown motif AV8.1-CAxGNNNARLMF-J31.1 paired with a unique cross-reactive motif BV3.2-CASSQALTDYGYTF-1.2. Once again this clone is most likely low affinity to IAV-M1 (i.e. BR tetramer+ in an IAV-M1 culture), but capable of proliferating during IAV infection resulting in a less than optimum functional response which is suggested by the fact that epitope-specific responses in older proliferated better than young, but had decreased ability to produce cytokines (**Supplemental Figure S1**).

## Discussion

Our study shows that IAV and EBV epitope-specific TCR repertoires change with increasing age and that TCR cross-reactivity likely plays a role in the repertoire changes between young and older donors. TCR high-throughput sequencing of tetramer-sorted epitope-specific and cross-reactive populations, and accessing TCR algorithms, such as, TCRdist (6), allowed us to obtain detailed information about TCR repertoire changes in not only TRBV/BJ, but also in TRAV/AJ usage. TCRa and TCRb repertoires directed at the HLA-A2-restricted immunodominant epitopes IAV-M1, EBV-BM and EBV-BR cognate and cross-reactive responses differed significantly between the older and young donors at every level we examined including CDR3 features, V and J usage and V/J pairing. Overall, these results strongly suggest that as the TCR repertoire narrows in older they are retaining TCR that are cross-reactive between these two very common human viruses IAV and EBV, that we are exposed to frequently, one with recurrent infections and the other a persistent virus, which frequently reactivates. For example, both high throughput sequencing and single cell sequencing suggest that a cross-reactive TCR clone AV27-CAGGGSQGNLIF-AJ-42 paired with BV19-CASSIRSSYEQY-JB2.7/2.1 previously considered to be a public clone in the IAV-M1 TCR repertoire (3) begins to dominate the EBV-BM and EBV-BR specific TCR repertoires in the older donors. This result suggests that the cross-reactivity with EBV-specific epitopes, leads to it being tweaked whenever EBV reactivates over a lifetime, making this clone so public that we have found it in the IAV-M1 repertoire of all the 40+ HLA-A2+ donors we have examined. However, these cross-reactive responses may not be optimal for control of one of these viruses. Cross-reactivity, with dual use of TCR may be the only alternative for an aging immune response (46), where the thymus has involuted and TCR repertoire keeps narrowing to help control a multitude of pathogens. This increased use of cross-reactive TCR may at some level save lives, but it may also contribute to the waning of virus-specific immunity with increasing age.

Our results suggest that with increasing age there is a preferential retention of TCR that have CDR3 features that increase their ease of generation (39–41) (44, 45), like the use of convergent recombinant amino acids and fewer N nucleotide additions, and cross-reactive potential by the use of glycine runs that are thought to be more flexible (42, 43) (47) (**Figures 2A–C**; summarized in **Supplemental Table S2**). Also, we were able to show that there were changes not only in TRBV, but also TRAV family usage, as well as, J family usage with increasing age for all three epitope specific responses. The TCRb high throughput sequencing data was consistent with the mAb staining data, in that there were fewer significant direct differences in TCRb usage than TCRa usage between young and older. If cross-reactivity is driving the change in TCR repertoire with increasing age this may arise from the fact that there is a great deal of overlap in BV usage between these epitope-specific responses. These data could be interpreted to suggest that perhaps TRAV usage may play a greater role in evolution of the TCR repertoire and in determining the specificity of TCR cross-reactivity further emphasizing the importance of studying TCRAV repertoire.

Here, we adapted the TCRdist program to analyze high throughput TRAV/AJ or TRBV/BJ sequences (**Figures 6**–**10**; **Supplemental Table S2**) were able to show there were differences in the pattern and specific TRAV/AJ and TRBV/BJ gene usage, pairing and CDR3 motifs in IAV-M1, EBV-BM and EBV-BR and cross-reactive responses of older and young donors. The cognate responses used public TCRa and TCRb features for all 3 epitope-specific repertoires, however, there were unique public features defined for the cross-reactive responses that differed from their cognate counterparts suggesting they are unique populations with their own characteristics, that make them capable of responding to two different antigens. The overlap in certain AV gene usages between epitopes, such as AV8 and AV12, would increase the potential for TCR cross-reactivity. Interestingly, AV12 has been found to be a public response in HLA-A2-restricted SARS-CoV2 YLQ epitope responses (48). As mentioned above, surprisingly, in the older donors the most dominant motif in the EBV-BM and EBV-BR TRBV repertoires was BV19-CASSIRSSYEQY-27, which known for being a public motif for IAV-M1. Overall, these results strongly suggest that as the TCR repertoire narrows in older donors they are retaining TCR that are cross-reactive between two very common human viruses IAV and EBV, that we are exposed to frequently, one with recurrent infections and the other a persistent virus, which frequently reactivates.

However, like the cognate repertoires, the cross-reactive M1BR and M1BM repertoires of older vs young donors differ suggesting that the older donors are retaining or developing a particular subset of cross-reactive T cells. Overall, these data suggest that young donors had most likely such private cross-reactive TCR repertoires that no motifs were identified for either M1BR or M1BM. In contrast, it would appear that older donors are most likely retaining selected cross-reactive TCR that have been stimulated by both antigens at some point leading to clonal expansions and identifiable public features. The results also suggest that a TCR that has some features of cognate responses may be more likely to be cross-reactive. However, these cross-reactive responses can also have totally unique public features, while displaying minor features if any of the cognate responses.

By using single cell sequencing we were able show some of the factors or features that may help a TCR to recognize two different epitopes. The single cell clones, further confirmed at a glance that the characteristics, including CDR3 motifs and distributions of the TCR clones are unique for each epitope and for each of the cross-reactive populations (**Figure 9**; **Supplemental Table S4**). They also further confirmed that the TRBV BV19/IRSS/J2.7 motif expressing clones, which are public for IAV-M1 repertoires were actually preferentially selected by cross-reactivity with the EBV epitopes. They also provided evidence that the cross-reactive clones had TCR features that would increase their ability to interact with two antigens. For instance, there is a clear selection for clones specifically expressing AV27-CAGGGSQGNLIF-AJ42 paired with BV19-CASSIRSSYEQY-JB2.7/2.1 in the M1+BR+, BRM1 and M1BM cross-reactive populations suggesting that this unique clone which dominates the EBV-BM and EBV-BR TCR repertoire of older donors has some ability to interact with all 3 epitopes (**Table 1A**). It may be at differing affinities to the different epitopes which might make it difficult to derive a crystal structure to determine exactly how it interacts with EBV-BM and EBV-BR (although it does appear to bind all three tetramers). If this type of clone which is most likely not optimum for EBV control begins to dominate the EBV-BM and EBV-BR TCR repertoires in older donors they may have difficulty controlling this persistent virus, enhancing chances of developing cancers. There is also a new unique cross-reactive TCR that predominates in the M1BM repertoire AV5-CAEPRDSNYQLIW-J33.1 paired with BV27-CASIGSGYPYNEQFF-2.1, where the AV5 family is public for EBV-BM and the motif could be a variant that could recognize EBV-BM, while the BV27 family has been shown in our studies to be used by all three epitope-specific responses, but this clone contains a CDR3b motif reminiscent of the public IAV-M1 BV19-xGxY-J2.1 (3) as shown in the **Table 1B**, which obligately pairs with AV38/AJ52 in the cognate IAV-M1 response. A second cross-reactive M1BM clone has a public IAV-M1-specific TCRa, AV27-CAGGGSQGNLIF-J42 paired with a public EBV-BM-specific TCRb, BV14-CASSQSPGGTGTF-J2.7. This clone is EBV-BM tetramer+ but able to proliferate in response to IAV-M1 peptide. Possibly without an appropriate TCRb, it is likely low affinity to IAV-M1 and unlikely to bind IAV-M1 tetramer, but could easily proliferate during an acute IAV infection, yet not be an optimum response to IAV.

The cross-reactive M1BR repertoire, had increased usage of clones expressing the public EBV-BR-specific AV8.1-CAVKDTDLIF-AJ34 (or variants of it) paired with multiple different TCRb chains some of which did express the IAV-M1 public BV19 family (**Table 1D**). Once again, as these M1BR clones are likely high affinity to EBV-BR, but low affinity to IAV-M1 it would not be ideal for them to start to proliferating during acute IAV infection. There was also a relatively unique public M1BR cross-reactive TCR which expressed the public EBV-BR AV8.1 with a relatively unknown motif AV8.1-CAxGNNNARLMF-J31.1 paired with a unique cross-reactive motif TCRb BV3.2-CASSQALTDYGYTF-1.2. This clone is most likely low affinity to IAV-M1 but capable of proliferating during IAV infection resulting in a less than optimum responses.

These studies highlight how important the develop of new tools and algorithms to study TCR repertoires, such as TCRdist and GLIPH (6, 29), can lead to our better understanding the evolution of antigen-specific repertoires. Other investigators are developing models that may assist us in predicting TCR specificity and cross-reactivity (28, 47–49). Developing more advanced computational methods for designing highly specific and potent TCR for use in engineering T cell therapies requires large amounts of accurate data on antigen-specific TCR repertoires and MHC-peptide complexes. Taken together all of these findings suggest that we have finally reached a paradigm shifting moment in our understanding of TCR structure and repertoire that could lead to a much better understanding of T cell mediated diseases and/or the development of T cell specific treatments.

Disease etiology and diagnosis by TCR repertoire analysis is beginning to gain more attention as technology improves (50). Several lines of evidence suggest that EBV-specific CD8 T cells are important for the control of EBV long term (51), including successful treatment of EBV-associated lymphoproliferative disorders and post-transplant associated EBV infections by adoptive transfer of EBV-specific CD8 T cells (52, 53). Recently, using high-throughput sequencing in multiple sclerosis (MS) patients, a disease associated with EBV-induced acute infectious mononucleosis, the TCR repertoire from the cerebrospinal fluid was found to be enriched in EBV-reactive CD8 T cells that were distinct from the blood^37^. TCR repertoires are increasingly being linked to disease (50) like recovery from cancer (54, 55), and our work which suggests that TCR repertoire differences contribute to protection against infection or impact disease severity (23, 24). Recent work (56) suggests that defective CD8 T cell control of EBV reactivation in multiple sclerosis (MS) patients leads to an expanded population of EBV-infected, autoreactive B cells; this is supported by preliminary results of a Phase I clinical trial that demonstrated improvement of MS symptoms following infusion of autologous EBV-specific CD8 T cells, which are thought to bring the virus under control (57). These types of T cell therapies make it imperative that more-advanced methods integrating computational biology and structural modeling become available for designing highly specific and potent TCRs. Methods to predict optimum TCR features to be recognized and activated by a particular antigen and for identifying TCR antigen-specificity groups without the need to isolate antigen-specific T cells would be highly valuable and are beginning to be developed (6, 29). Recently, progress has been made in developing algorithms that identify crossreactive epitopes, between strains of similar viruses like IAV and coronaviruses (58).

These results also impact our understanding of the current COVID19 pandemic, where a disease caused by the severe acute respiratory syndrome coronavirus-2 (SARS-CoV-2), can present in many forms. It generally causes a mild and sometimes asymptomatic disease in children but is more pathogenic in adults and can be quite severe in aged populations, especially in individuals with pre-existing conditions. Yet, individuals of similar age and health status may experience widely different disease processes and severity. In severe cases lungs may encounter a highly inflammatory cytokine storm and be full of CD8 T cells experiencing various degrees of clonal exhaustion (59). Reasons for the variation in pathogenesis are unknown and could be influenced by viral dose and genetics of the host, but it is likely that T cell-dependent heterologous immunity and cross-reactivity play a role (60). In mouse models, virally induced pathologies have been linked to cross-reactive epitopes and can vary widely among individuals, even those with similar genetics and infection histories. In several models with syngeneic hosts, variability in the pathogenesis has been linked to the private specificity of the T cell repertoire responding to the cross-reactive epitope (17, 18, 25). Even genetically identical hosts have different naïve TCR repertoires as well as different TCR repertoires to the same epitope of a pathogen. These results suggest that how an individual would respond to SARS-CoV-2 would be dependent in part on that person’s TCR repertoire and history of previous infections.

Heterologous immunity is likely common in human virus infections, and, like COVID-19 pathogenesis, there are several common human viruses that cause more severe disease in adults than in children. Here, the disease in adults is usually associated with more immunopathological lesions. These include such viruses as measles, mumps, chicken pox, and EBV. Like SARS-CoV2, EBV causes mild to subclinical infections in children, but it can cause acute infectious mononucleosis (AIM) in young adults. AIM, in HLA-A2+ individuals, is associated with a high frequency of CD8 T cells producing high levels of interferon gamma and being cross-reactive between EBV and IAV (23, 26, 61). In fact, the severity of AIM directly correlated with frequency of reactivated IAV-M1_58_ tetramer+ cells and its’ TCRBV usage. The main pathognomonic feature of AIM is the potent CD8 T cell response, much like that occurring in lungs of severe COVID-19 cases (62). Interestingly, a recent report examining TCR repertoires suggest that in HLA-A2+ patients certain SARS-CoV-2 epitope-specific responses are also cross-reactive with IAV epitopes and in they have been found to use the TRBV sequence ‘CASS(I/x)RS(T/A/S)EQYF” (63). This suggests that prior immunity to IAV can predispose hosts to severe EBV infection, but may also effect SARS-CoV-2 infection outcome. In an attempt to model this in the absence of EBV infection, mice immune to IAV were challenged intranasally with lymphocytic choriomeningitis virus (LCMV). These mice developed a severe pneumonia that was dependent on cross-reactive CD8 T cell responses to either of two epitope pairs, depending on the private specificity of the response. Interestingly, the development of this pneumonia was blocked by injecting IAV-immune mice with antibody to IFNg prior to the LCMV challenge (19)

These similarities between COVID-19 pathogenesis and heterologous immunity would suggest that there may be cross-reactive epitopes between SARS-CoV-2 and previously encountered infections, though it is usually hard to predict where cross-reactivities would occur. However, humans get infected with a number of other coronaviruses that cause common colds and serologically cross-react with SARS-CoV-2, providing a challenge for the development of antibody screening tests. Further, studies using an algorithm for T cell epitopes have predicted many potential cross-reactivities across a variety of class 1 MHC molecules (64–66). Other recent reports document some CD4 and CD8 T cell cross-reactivity between SARS-CoV-2 and other coronaviruses (67–70). One of these studies suggests that if there is a broader cross-reactive epitope usage the patients may be more likely to have milder disease (68). Another study actually correlates severity of acute COVID to specific cross-reactive TCR repertoires between coronaviruses in HLA-A-2+ patients (70). If such cross-reactivity exists, it may be an issue in the development of the much-needed vaccines for SARS-CoV-2. However, the presence of cross-reactive T cell epitopes in complex vaccines may lead to high variability in the outcomes (21). Further, when epitopes cross-react only partially with a memory T cell pool specific to another epitope, the T cell response that develops may be very narrow and oligoclonal, with a potential to allow for T cell-escape mutants (61). The presence of a narrow oligoclonal repertoire like we see in the older donors to a T cell epitope during an acute infection is likely a product of this cross-reactivity process. Interestingly, a recent study showed that exposure order determined the distribution between spike-specific and non-spike-specific responses in COVID19 CD8 T cell response (71). Vaccination after infection lead to expansion of spike-specific T cells and differentiation to CCR7(neg)CD45RA(pos) effectors. In contrast, individuals having a breakthrough infection after vaccination, developed vigorous non-spike-specific responses. Their extensive epitope-specific T cell antigen receptor (TCR) sequence analyses showed that all exposures elicited diverse repertoires characterized by shared public TCR motifs, with no evidence for repertoire narrowing from repeated exposure (71). Given our present results and all of these issues we suggest that the examination of T cell cross-reactivity and TCR repertoire should be given high priority in COVID-19 research.

## Data availability statement

The original contributions presented in the study are publicly available. This data can be found here: https://www.ncbi.nlm.nih.gov/sra/PRJNA928775.

## Ethics statement

The studies involving human participants were reviewed and approved by The Institutional Review Board (IRB) at University of Massachusetts Medical School, Worcester, MA, USA. The patients/participants provided their written informed consent to participate in this study.

## Author contributions

FC, AG, IT, NA, DG and LSK contributed to literature review. FC, AG, LS contributed to writing. FC, AG, IT, NA, DG and LKS were responsible for editing. AG and LS oversaw the writing process and provided mentorship and guidance. All authors contributed to the article and approved the submitted version.

## Supplementary material

Supplementary Figure 1 Function of IAV-M1- and EBV-specific CD8 T cell responses differ between young (Y) and older (O) donors. Functionality was assessed by examining antigen-specific proliferation using tetramer staining in short-term culture **(A-C)** and by cytokine production **(D)**. CD8 T cells were cultured and re-stimulated for 3 weeks in the presence of T2-cells pulsed with specific peptides, IAV-M1, EBV-BM, EBV-BR, and tyrosinase, a self-peptide derived from melanocytes as a control. Representative FACS plot of a young **(A)** and an older donor **(B)** shows antigen-specific proliferative capacity as measured by tetramer staining in short-term culture. The upper row is single tetramer staining specific to the stimulating peptide (cognate) and the lower row is co-staining with two tetramers showing proliferation of cross-reactive CD8 T cell responses in IAV-M1-stimulated short-term cultures. In both O and Y donors IAV-M1 stimulation in culture resulted in the proliferation of two types of IAV-M1 and EBV cross-reactive responses as we have previously reported (23, 61), IAV-M1+EBV-BR+ (M1+BR+) and IAV-M1+EBV-BM+ (M1+BM+) tetramer co-staining CD8 T cells or EBV-BR (M1BR) or EBV-BM (M1BM) single tetramer staining cells. C) Older donors had higher frequencies of EBV-BM and EBV-BR than young donors as shown in a heatmap of mean tetramer frequencies (Y n=8-11; O n=7-9). For the cognate (same as peptide used to stimulate culture) responses, in Y the IAV-M1 tetramer frequency was significantly greater than EBV-BR (p=**); in older donors the EBV-BM tetramer frequency was significantly greater than IAV-M1 (p=****) and EBV-BR (p=****). D) Y donors had higher frequencies than older donors of IFNγ+MIP1beta+ cytokine-producing CD8 T cells in IAV-M1 (i), EBV-BM (ii) and EBV-BR (iii) stimulated short-term cultures. Short-term cultured CD8 T cells were stimulated with indicated peptides. Controls were PMA and Ionomycin or no peptide stimulation (gated on cognate tetramer+ cells). Multi-variant 2-way ANOVA with adjusted p-value, *p < 0.05, **p < 0.01, ***p < 0.001, ****p < 0.0001. The specificity of the culture is represented by a unique color, IAV-M1 is red, EBV-BM turquoise blue, and EBV-BR dark blue, these are retained throughout the manuscript. These results suggest that there are functional differences in EBV-specific responses between these two age groups. It would appear that the EBV-specific responses have greater proliferative capacity, but decreased ability to produce cytokines such as IFNγ and MIP1β in older donors. Interestingly, the IAV-M1 proliferative capacity and the ability to induce cross-reactive responses with EBV does not differ between young and older.
