# Supplementary figures and images for: Cross-reactivity influences changes in human influenza A virus and Epstein Barr virus specific CD8 memory T cell receptor alpha and beta repertoires between young and old
Source: Front Immunol. 2023 Feb 24;13:1011935. doi: 10.3389/fimmu.2022.1011935 (PMC10009332; doi:10.3389/fimmu.2022.1011935)

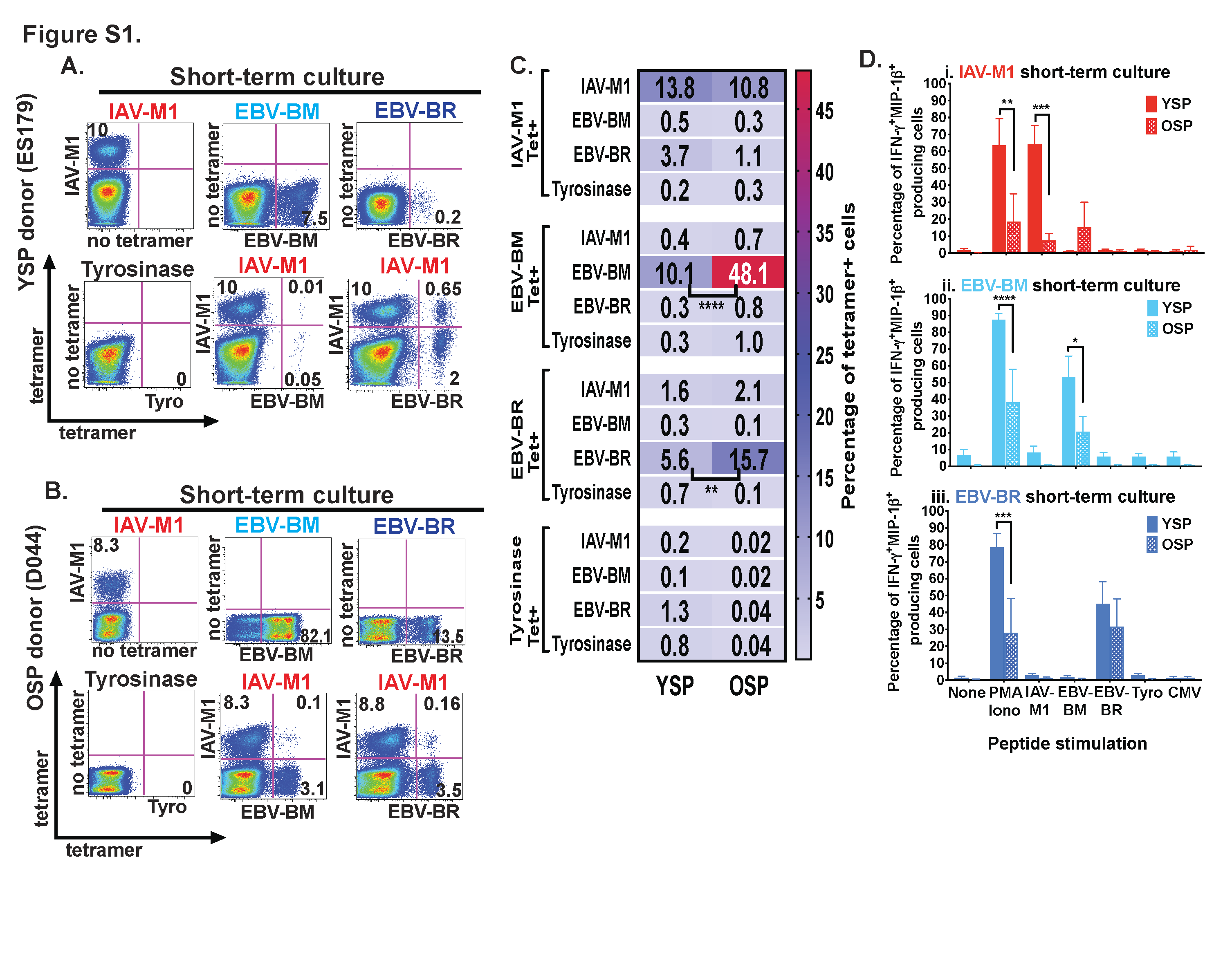

Supplement: Supplementary Figure 1 — Function of IAV-M1- and EBV-specific CD8 T cell responses differ between young (Y) and older (O) donors. Functionality was assessed by examining antigen-specific proliferation using tetramer staining in short-term culture (A-C) and by cytokine production (D). CD8 T cells were cultured and re-stimulated for 3 weeks in the presence of T2-cells pulsed with specific peptides, IAV-M1, EBV-BM, EBV-BR, and tyrosinase, a self-peptide derived from melanocytes as a control. Representative FACS plot of a young (A) and an older donor (B) shows antigen-specific proliferative capacity as measured by tetramer staining in short-term culture. The upper row is single tetramer staining specific to the stimulating peptide (cognate) and the lower row is co-staining with two tetramers showing proliferation of cross-reactive CD8 T cell responses in IAV-M1-stimulated short-term cultures. In both O and Y donors IAV-M1 stimulation in culture resulted in the proliferation of two types of IAV-M1 and EBV cross-reactive responses as we have previously reported (23, 61), IAV-M1+EBV-BR+ (M1+BR+) and IAV-M1+EBV-BM+ (M1+BM+) tetramer co-staining CD8 T cells or EBV-BR (M1BR) or EBV-BM (M1BM) single tetramer staining cells. C) Older donors had higher frequencies of EBV-BM and EBV-BR than young donors as shown in a heatmap of mean tetramer frequencies (Y n=8-11; O n=7-9). For the cognate (same as peptide used to stimulate culture) responses, in Y the IAV-M1 tetramer frequency was significantly greater than EBV-BR (p=**); in older donors the EBV-BM tetramer frequency was significantly greater than IAV-M1 (p=****) and EBV-BR (p=****). D) Y donors had higher frequencies than older donors of IFNγ+MIP1beta+ cytokine-producing CD8 T cells in IAV-M1 (i), EBV-BM (ii) and EBV-BR (iii) stimulated short-term cultures. Short-term cultured CD8 T cells were stimulated with indicated peptides. Controls were PMA and Ionomycin or no peptide stimulation (gated on cognate tetramer+ cells). Multi-variant 2-way ANOVA with [file Image_1.tiff]

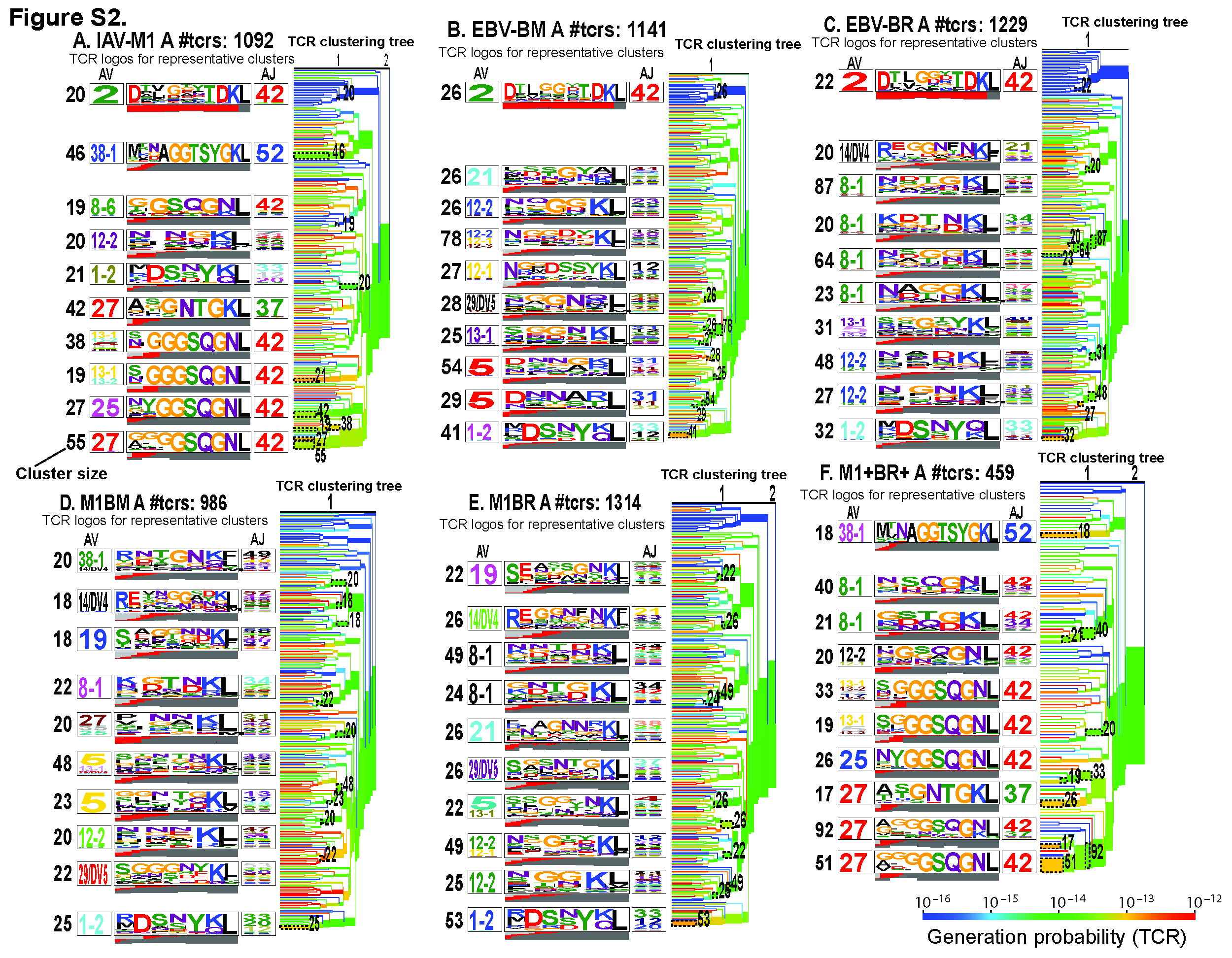

Supplement: Supplementary file 2 [file Image_2.tiff]

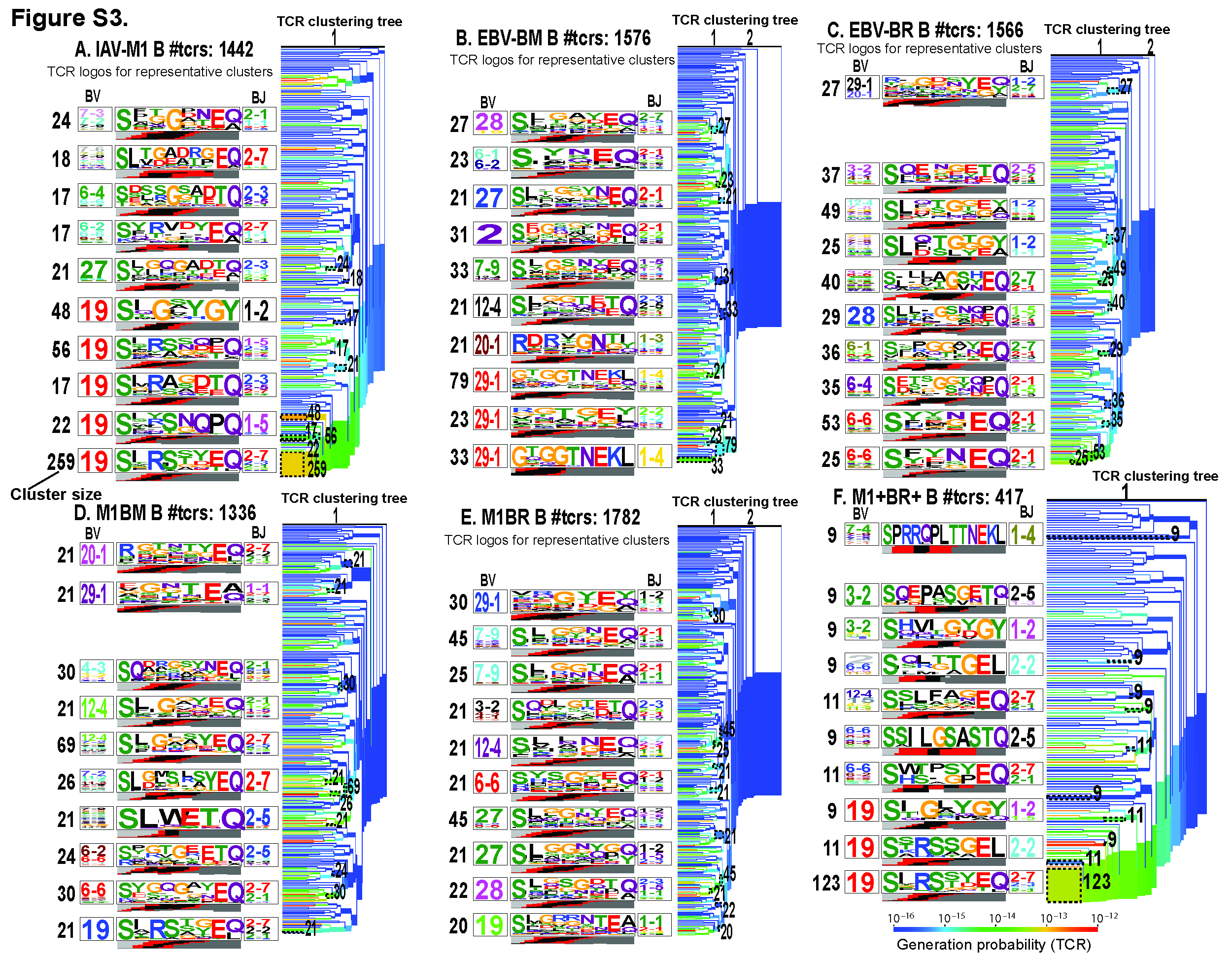

Supplement: Supplementary file 3 [file Image_3.tiff]

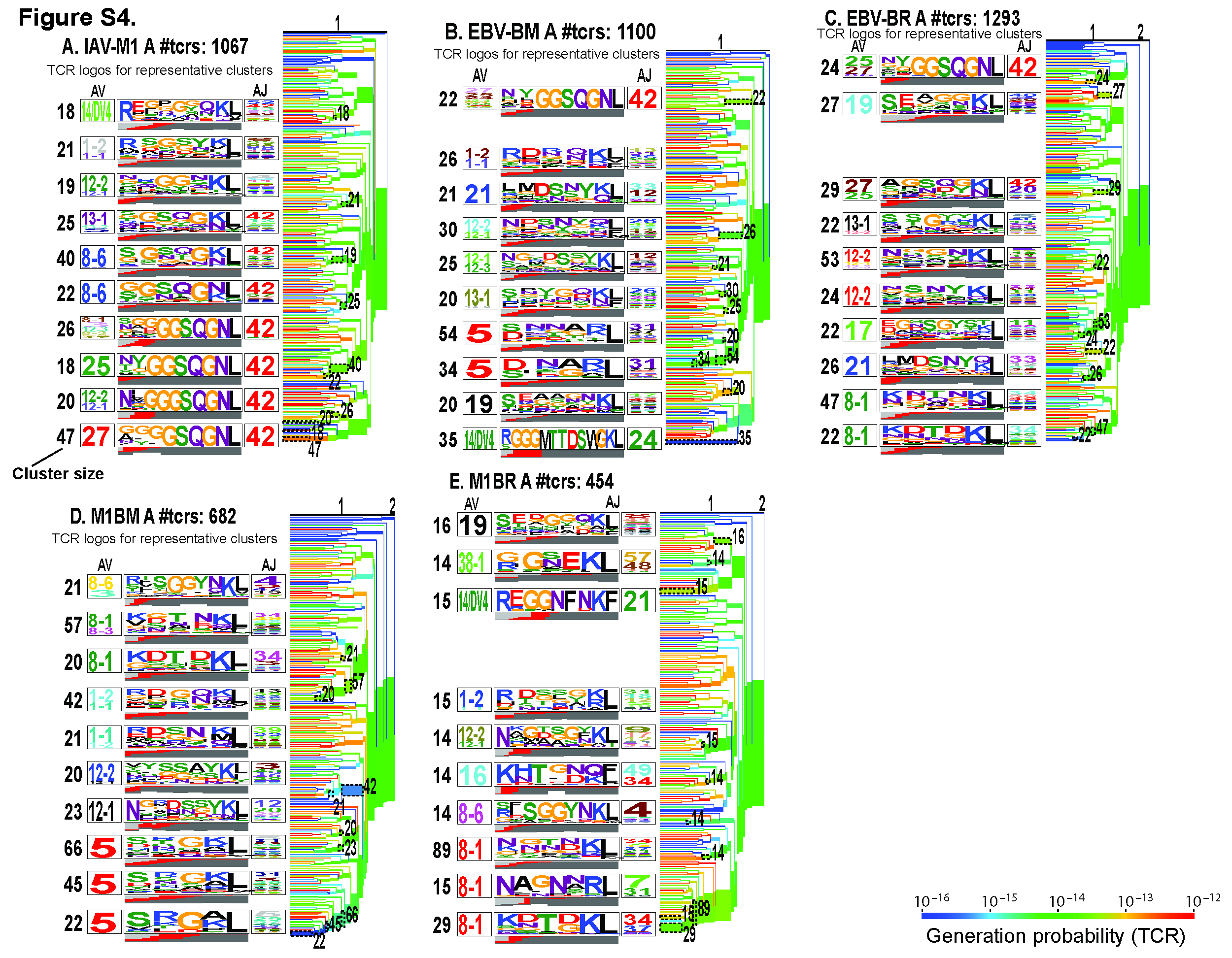

Supplement: Supplementary file 4 [file Image_4.tiff]

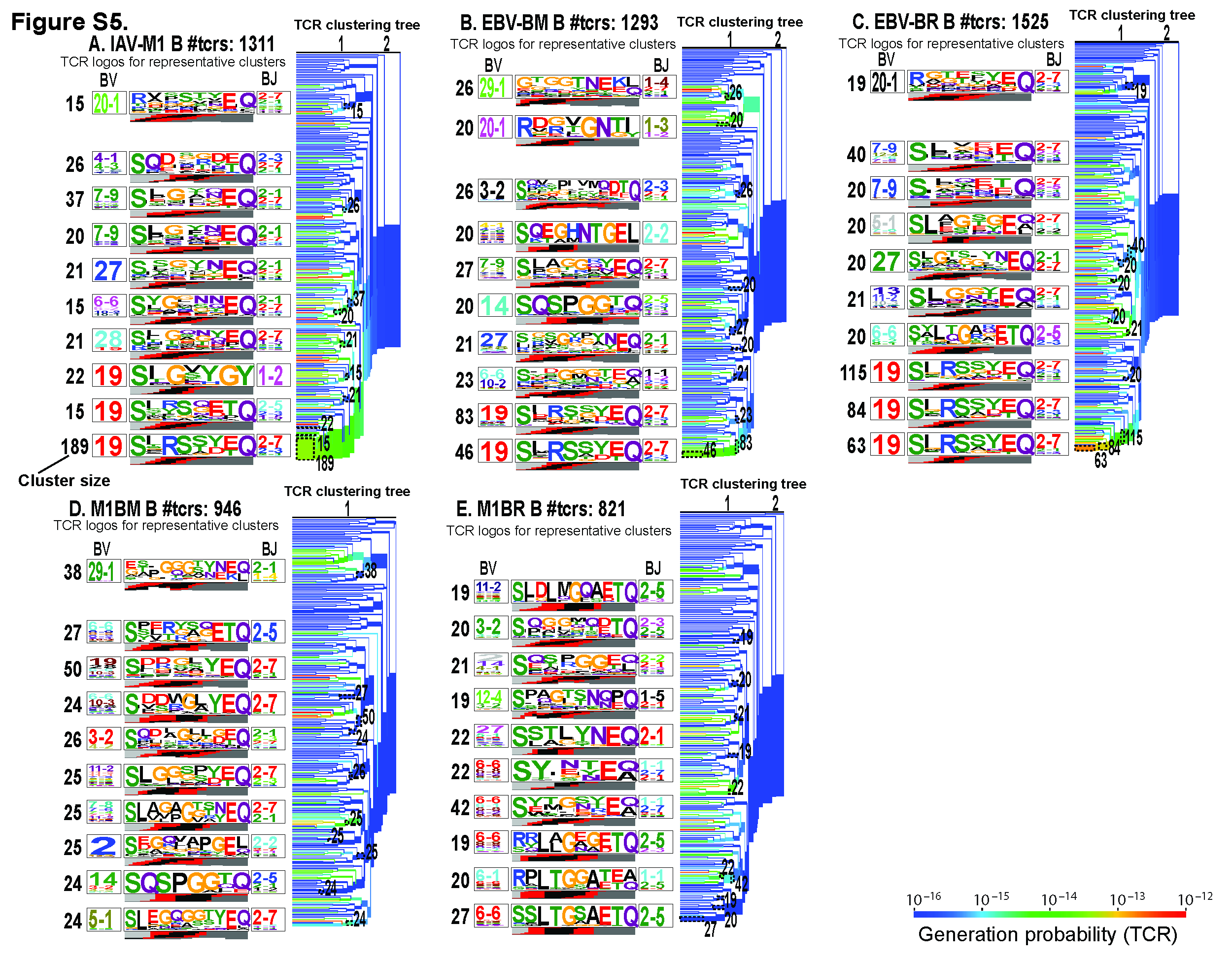

Supplement: Supplementary file 5 [file Image_5.tiff]
