# Supplementary material for: Cross-reactivity influences changes in human influenza A virus and Epstein Barr virus specific CD8 memory T cell receptor alpha and beta repertoires between young and old
Source: Front Immunol. 2023 Feb 24;13:1011935. doi: 10.3389/fimmu.2022.1011935 (PMC10009332; doi:10.3389/fimmu.2022.1011935)
Supplement: Supplementary file 6 [file Table_1.pdf]

Table S1 A. Study Population used for TCR high throughput sequencing

Young (Y) Donors	Age	Gender
ES179	18	Male
ES181	18	Female
ES556	19	N/A
D115	22	Female
**mean±SEM**	**19±1**	
**Older (O) Donors**		
CT044	72	Female
D002	66	Female
D035	68	Male
D044	66	Female
D05	83	Male
**mean±SEM**	**71±4**	
Note: The “N/A” stands for not available		

Table S1B. Study Population used for monoclonal antibody (mAb) stainings

Young (Y) Donors	Age	Gender
ES179	18	Male
ES180	19	Male
ES181	18	Female
ES194	19	Male
ES236	19	Female
ES556	19	N/A
ES587	19	Female
ES601	20	Female
ES640	18	Female
D115	22	Female
**Mean**	**19±1 (SEM)**	
**Older (O) Donors**	**Age**	**Gender**
CT044	72	Female
CT050	74	Male
D002	66	Female
D035	68	Male
D044	66	Female
D04	82	Female
D05	83	Male
HD003	70	Female
HD007	73	Female
HD008	64	Male
**Mean**	72±3 (SEM)	

Note: The “N/A” stands for not available.
