# Supplementary material for: Cross-reactivity influences changes in human influenza A virus and Epstein Barr virus specific CD8 memory T cell receptor alpha and beta repertoires between young and old
Source: Front Immunol. 2023 Feb 24;13:1011935. doi: 10.3389/fimmu.2022.1011935 (PMC10009332; doi:10.3389/fimmu.2022.1011935)
Supplement: Supplementary file 7 [file Table_2.docx]

| TRAV  **TABLE S2. Summary of features of IAV-M1, EBV-BM, EBV-BR, M1BR and M1BM TCRA (A and B), TCRB (C and D) repertoires in OSP and YSP.** | IAV-M1 |  | EBV-BM |  | EBV-BR |  | M1BR |  | M1BM |  |
| --- | --- | --- | --- | --- | --- | --- | --- | --- | --- | --- |
|  |  |  |  |  |  |  |  |  |  |  |
| A. | **OSP vs. YSP** | | **OSP vs. YSP** | | **OSP vs. YSP** | | **OSP vs. YSP** | | **OSP vs. YSP** | |
| i. No. glycines |  | | **OSP**>YSP | | **OSP**>YSP | | OSP<**YSP** | |  | |
| ii. No. nucleotides |  | | **OSP**>YSP | |  | |  | |  | |
| iii. No. RAA |  | | **OSP**>YSP | | **OSP**>YSP | |  | |  | |
| iv. AV usage  (Heatmap) | **OSP**>YSP: **AV8**  OSP<**YSP**: AV38 | | **OSP**>YSP: **AV5, AV29** | | **OSP**>YSP: **AV21**  OSP<**YSP**: AV12 | | OSP<**YSP**: AV21 | | **OSP**>YSP: **AV5, AV12** | |
| v. AJ usage  (Heatmap) | OSP<**YSP**: AJ 51.1, AJ58.1 | | **OSP**>YSP: **AJ45.1**  OSP<**YSP**: 20.1 | |  | | OSP<**YSP**: 31.1 | | **OSP**>YSP: **AJ34.1, AJ49.1**  OSP<**YSP**: AJ26.1 | |
| B. | **OSP** | **YSP** | **OSP** | **YSP** | **OSP** | **YSP** | **OSP** | **YSP** | **OSP** | **YSP** |
| i. CDR3 length | **10-mer**, 12-mer | 13-mer,12-mer | 11-mer | 11-mer,10-mer | **12-mer** | 9-mer | **None** | 12-mer,  10-mer, 9-mer | 12-mer | 12-mer |
| ii. AV family >10%  (Heatmap) 5-10% | **AV27, 8,** 13  38, 12 | **AV38**  27, 13, 41 | **AV5**, 29  12, 8 | **AV5, 12**  8, 13 | **AV8**, 21  12, 14, 24 | **AV12, 8**  13, 17 | **AV8**  16, 14, 12, 5 | **AV8**, 21  16, 12 | **AV5**, 8, 12 | 38,25, 41,5 29 |
| iii. AJ family >10%  (Heatmap) 5-10% | **AJ42**  18, 52 | **AJ42**, 51  58, 52 | AJ45, 42, 12, 49 | **AJ20**  31, 12, 33 | **AJ34**  33, 21 | **AJ34**  27, 21, 53 | 21,34,49,15,26,31 | **AJ34,** 31  DJ01 | **AJ49**, 34, 4  33 | **AJ26**,  52 |
| iv. AV gene:  (ribbon plots: top 400 clonotypes)  -bold: differences between OSP and YSP  -colors: BV usage overlap in more than 4 specificities  - fold increase | 27- 4x  **12.2- 2x**  **8.6- 2x**  38.1  **24- 2x** | 27- 4x  **2**  38.1  **19** | 5- 2x  **14/DV4**  21  12.2- 2x | 5- 4x  **2- 2x**  12.2- 2x  21  **1.2- 2x** | 12.2- 2x  8.1- 2x  **21**  **14/DV4**  **24- 2x** | **2- 2x**  8.1- 4x  12.2- 2x  **1.2** | **8.1- 8x**  **14/DV4**  1.2  **16- 2x**  **8.6- 2x** | **2- 2x**  **12.2- 2x**  1.2- 2x  **21** | 5- 4x  8.1- 2x  **12.2**  1.2- 2x  **1.1- 2x** | **29/DV5**  **38.1**  **19**  1.2- 2x  8.1  5- 2x |
| v. AJ gene:  (ribbon plots: top 400 clonotypes)  (same as above for bold and colors, and fold increase) | 42- 2x  37  52  **43** | 42- 4x  37  52  **33** | 42  **24- 2x**  **26**  33  11- 2x  12- 2x | 42- 2x  **20- 2x**  **31**  33  **30- 2x**  12- 2x  11- 2x | 42- 2x  **43**  **40**  **34**  **11- 2x** | 42- 2x  **34- 2x**  **20**  **33**  **12- 2x** | **34- 2x**  **21- 4x**  **37- 2x**  **49** | **42**  **33**  **20**  **31**  **3- 2x**  **11- 2x**  **12- 2x** | 42  **45**  **33- 2x**  **48**  **13- 2x**  **3- 2x**  **12- 2x** | 42  **20**  **52**  **34** |
| vi. AV/AJ gene pairs  (ribbon plots: top 400 clonotypes for each donor)  (same as above for bold and colors, and fold increase) | V27/J42- 3.2x  V38/J52- 8.1x  V25/j42- 3.0x | V27/J42- 1.7x  **V2/J42- 3.4x**  V38/J52- 11x  V25/J42- 2.6x  **V1.2/J33- 7.8x**  **V1.2/J12- 13x**  **V27/J37- 4.8x** | V2/J42- 6.6x  **V5/J31- 5.4x**  **V14/DV4/J24-7.2x** | V2/J42- 8.1x  **V5/J37- 5x**  **V1.2/J31- 6.5x**  **V1.2/J12- 5.5x**  **V12.1/J12- 6.8x**  **V12.3/J52- 18.5x** | V2/J42- 6.9x  **V27/J42- 5x** | V2/J42- 7.8x  **V1.2/J33- 6.8x**  **V29/DV5/J52- 9.4x**  **V27/J11- 13.8x** | V14/DV4/J21-6.9x  **V16/J49- 10.9x**  **V8.6/J -18.9x**  **V38.1/Jx -24.8x**  **V17/Jx -20.6x** | **V2/J42- 9x**  V14/DV4/J21- 7x  **V1.2/J33- 5.3x**  **V1.2/J12- 7.3x**  **V17/J11- 10x** | V2/J42- 9.3x  **V8.3/J49- 9.8x**  **V8.6/J4- 9.8x** | V2/J42- 6.8x  **V19/J33- 8.2x**  **V17/J11- 8.8x** |
| vii. CDR3 motifs:  motif 1 shown  (motif 2 shown in red)  ( top 400 clonotypes) | **V12.1/12.2/8.1-NxGGGSQ**-J42;  V27/**12.2/8.1**/25-NGGGSQG-J42; | **V38-FMx(N/G/D)AGGT-J52;**  V27/**13.1**/25-GGGSQ-J42 | **V14/DV4-CAMRGGGMT-J24;**  V5**/6/2**-CAEDxNARLM-J31 | V5-CA(E/D)DxNARLM-J31 | **No motif** | **V8.1/8.3/16/12.2-VKDTDKL-J34/37** | **V8.1/16/12.2-CA(V/L)K(D/N)TD**  **KLIF-J34/37** | **No motif** | **V5-CAES(R/K/T)GxLxF-J5/29/37/41;**  **V8.1/16/1.1-**  **CAVKDTDKLI-J34/23;**  **V5/8.6/8.4-**  **CAED-J31/5/33** | **No motif** |

| TRBV | IAV-M1 |  | EBV-BM |  | EBV-BR |  | M1BR |  | M1BM |  |
| --- | --- | --- | --- | --- | --- | --- | --- | --- | --- | --- |
| C. | **OSP vs. YSP** | | **OSP vs. YSP** | | **OSP vs. YSP** | | **OSP vs. YSP** | | **OSP vs. YSP** | |
| i. No. glycines | **OSP**>YSP | | **OSP**>YSP | | **OSP**>YSP | | **OSP**>YSP | | **OSP**>YSP | |
| ii. No. nucleotides | OSP<YSP | | OSP<YSP | | OSP<YSP | | **OSP**>YSP | | **OSP**>YSP | |
| iii. No. RAA |  | | **OSP**>YSP | | **OSP**>YSP | |  | |  | |
| iv. BV usage  (Heatmap) |  | |  | | **OSP**>YSP: **BV10**  OSP<**YSP**: BV6 | |  | |  | |
| v. BJ usage  (Heatmap) |  | |  | |  | |  | | **OSP**>YSP: **BJ2.7** | |
| D. | **OSP** | **YSP** | **OSP** | **YSP** | **OSP** | **YSP** | **OSP** | **YSP** | **OSP** | **YSP** |
| i. CDR3 length | 11>13 | 11>13>12 | 11>13>12 | 13>11>12>14 | 13>12>11>14 | 13>12>11 | **13** | 11>12>13 | **14>12** | 11 |
| ii. BV family >10%  (Heatmap) 4-10%  (large most dominant) | BV19  **5**,18,7,**29** | BV19  18, **6**, 7,**27** | BV14, **27**, 10  **4**, 29, 20, 2 | BV29, 14**, 3**, **7**  20, **9**, 2,10 | **BV10**, 7, 3, 19, 28, **29**, **20**, 6, 4, 5, **27** | BV6**, 24,** 28, 3, 5, 4, **14**, 19, 7, **12** | BV3, 6, **12**, **27**, **21**, **29**, 5, **19** | BV3, 6, **4**  **7**, **28**, **25**, **13**, 5, **24** | **BV10**, **28**, 27, 14, 7, **29**, **3**, **2**, 19 | BV19,  7, **4**, **6**, 27 |
| iii. BJ family >10%  (Heatmap) 4-10%  (bold most dominant) | **BJ2.7**, 2.1, 2.3,  2.5, 1.2 | **BJ2.7, 1.2,** 2.1,2.3  1.5, 1.1, 2.2 | **BJ2.7,2.3,**2.1,2.2 | **BJ2.1, 2.4,** 2.7  1.2, 1.4, 1.5, 1.1 | **BJ1.1,2.1,**2.7, 2.5,2.3, 2.2, 1.2 | **BJ2.1**, 2.5,2.7, 1.2  2.3, 1.1 | **BJ2.1**,1.5,2.5,2.7  1.2 | **BJ2.1,** 1.2  1.1,2.5,2.7,2.3,1.5 | **BJ2.7**, 2.1, 2.2,2.5 | **BJ2.1**, **1.2**  2.7, 2.5, 2.2 |
| iv. BV gene:  (ribbon plots: top 400 clonotypes)  -bold indicates differences between OSP and YSP  -colors indicate BV usage overlap in more than 4 specificities  - fold increase above naïve indicated | 19- 4x  7.9  27  **28**  6.6  **3.2**  **21.1- 4x** | 19- 4x  27  **2.4**  6.6  **20.1**  7.9  **6.4- 2x** | **19**  7.9  27  6.6  **20.1**  **3.2**  10.2- 4x  14- 2x  **4.1- 2x**  21.1- 8x | **29- 2x**  7.9  27  6.6  **28**  **9**  **6.4- 2x**  14- 2x  21.1- 2x  10.2- 2x | **19- 2x**  27  7.9  6.6  3.2  **20.1**  **10.2- 4x**  **21.1- 4x** | 6.6  27  **28**  **12.4**  3.2  **29**  **6.4- 4x**  **7.8- 2x**  **13- 2x**  **14- 2x** | 6.6  3.2- 2x  29  **6.1**  27  **20.1**  21.1- 16x | 6.6  27  29- 2x  **7.9**  **28**  3.2  **6.4- 2x**  **13- 2x**  **14-2x**  **10.2- 4x** | **3.2- 2x**  **29- 2x**  **2**  6.6  20.1  **28**  **11.2- 2x**  14- 4x  21.1-16x | 6.6  **27**  19  **4.3**  20.1  **7.9**  **11.3- 2x**  **10.2- 4x**  14- 2x  21.1-16x |
| v. BJ gene:  (ribbon plots: top 400 clonotypes)  (same as above for bold and colors, and fold increase) | 2.7  2.1  2.3  **2.5**  **2.6- 2x** | 2.7  2.1  2.3  **1.1** | 2.7  2.1  2.3  **2.2**  **2.6- 2x** | 2.1  2.7  2.3  **1.2** | 2.7  2.1  **2.3**  1.1  **1.6- 2x** | 2.1  2.7  **1.2**  1.1 | 2.1  **2.5**  2.7  1.1 | 2.1  1.1  **2.3**  2.7 | 2.7  2.1  2.5  **2.2** | 2.7  2.1  2.5  **2.3** |
| vi. BV/BJ gene pairs  (ribbon plots: top 400 clonotypes)  (same as above for bold, colors, and fold) | V19/j2.7- 1.5x | V19/j2.7- 1.4x  **V19/J1.1-0.4x** | **V19/j2.7- 2.2x**  **V20.1/J1.3- 7.1x**  **V3.2/J1.4- 7.9x**  **V2/J2.2- 4.5x** | **V29/J1.4- 5.1x**  **V20.1J2.6- 6.0x** | **V28/J1.5- 4.6x**  **V10.2/J1.1- 5.0x** | NS | **V6.6/J2.5- 2.7x**  **V11.2/J2.5- 4.8x** | **V6.4/J2.3- 3.7x** | **V29/J1.4- 6.7x**  **V2/J2.2- 4.9x**  **V20.1/J1.3- 8.6x**  **V5/2.7-2.9x** | NS |
| vii. CDR3 motifs: motif 1 shown  (motif 2 shown in red)  (ribbon plots: top 400 clonotypes) | **V19**-**CASSIRSSYEQYF**-**J2.7**/2.3/2.1;  V7.7/7.3/6.6//5.6/10.3/10.2/21.1-  **QSRANVLTF-J2.6;**  **V4.1**/17.9/7.2/5.8/5.4/4.3/4.2-SSQDWTGNTDT**-J2.3** | **V19-CASSIRSSYEQYF**-**J2.7**/2.3/2.1/2.2/  2.5/1.5;  **V6.2/6.7/6.6**/3.2/  28/27/12.4-  SY(P/S)(R/D)(T/R)GHSNQP-**J1.5** | V7.7/7.3/6.6//5.6/10.3/10.2/21.1-  **QSRANVLTF-J2.6;**  **V19**-**CASSIRSSYEQYF-J2.7**;  **V14**/3.2/(N=21BV)-QS(R/P)(A/G)-**J2.6** (N=9BJ)  (5 MORE MOTIFS) | **V14/23.1/9/7.7/6.2**/3.2/7.4/6.7/5.7/5.3/4.2/10.3-  QS(R/P)(A/G)(N/G)(V/I)(L/Q)(T/Y)F-  **J2.6**/2.4/2.7/2.3/  2.1 | **V19**-**CASSIRSSYEQYF**-**J2.7**;  **V10.2**/4.3/10.1-  CASSxDGMNTEA-**J1.1** | **V6.6**/6.4/(N=21BV)-CASSF(E/Y/P)NE-**J2.1**/2.5;  V5.6/7.9/7.2/6.4/4.3/27/11.2/6.2/5.5/  5.1/28-  IG(S/T)GEL-**J2.2**  **V6.6/9/6.**4/6.2/6.1/3.2/28/27/12.4-  SFPGGAN-**J2.1**  (5 MORE MOTIFS ) | **V6.6**/6.1/5.6/11.2/  10.3 (N=24 BV)-  CAS(S/R)(P/S)(L/F)(T/S)G(S/G)A(E/D/T)TQYF- **J2.5**/2.3/1.1  **V3.2/**7.9/6.6/5.8/5.4/5.1/4.1/30/19/12.4/11.3-  KTYGY-**J1.2**  V7.6/7.3/7.7/7.4  7.2/7.1/6.2/4.1/3.2/29.1/2/12.5-  **QSRANVLTF-J2.6;**  (10 MORE MOTIFS) | no motif | **V14**/3.2/4.2/18/21.1/7.8/6.1-  ASSQSPGG-J2.5/2.1/1.2/2.6/2.4  **V2**/27/7.7/13/12.4-  SS(D/E)G(Q/K)/V/L/I)(A/L/F)PGELF-  **J2.2**/2.1  V6.6/7.8/7.7/7.4/7.3/7.2/7.1/6.2/19/12.4/11.2/10.1/1-  **QSRANVLTF-2.6**  (13 MORE MOTIFS) | V6.6/7.9/6.1/7.7/7.8/7.7/7.4/  7.3/7.1/7.2 (N=19BV)-  ASSLWETQY-**J2.5**/2.7  **V7.2**/7.9/7.4/7.3/7.1/4.3/14/11.3/11.2-  CASSPMRG-**J2.3**/2.5/1.1  V12.4/7.8/7.3/1/7.4/7.2/6.2/4.1/24.1/11.3  **QSRANVL-2.6**  (4 MORE MOTIFS) |
